# Microarray expression profile of mRNAs and long noncoding RNAs and the potential role of PFK-1 in infantile hemangioma

**DOI:** 10.1186/s13008-020-00069-y

**Published:** 2021-01-11

**Authors:** Kaiying Yang, Xuepeng Zhang, Linwen Chen, Siyuan Chen, Yi Ji

**Affiliations:** 1 Division of Oncology, Department of Pediatric Surgery, West China Hospital of Sichuan University, #37 Guo-Xue-Xiang, Chengdu, 610041 China; 2 Pediatric Intensive Care Unit, Department of Critical Care Medicine, West China Hospital of Sichuan University, Chengdu, 610041 China; 3 College of Clinical Medicine, North Sichuan Medical College, Nanchong, Sichuan 637000 China

**Keywords:** Infantile hemangioma, Long noncoding RNA, mRNA, PFK-1, Microarray, Bioinformatics analysis

## Abstract

**Background:**

Infantile hemangioma (IH) is the most common benign tumor in children. Long noncoding RNAs (lncRNAs) play a critical role in tumorigenesis. However, the expression levels and biological functions of lncRNAs in IH have not been well-studied. This study aimed to analyze the expression profile of lncRNAs and mRNAs in proliferating and involuting IHs.

**Methods:**

The expression profiles of lncRNAs and mRNAs in proliferating and involuting IHs were identified by microarray analysis. Subsequently, detailed bioinformatics analyses were performed. Finally, quantitative real-time polymerase chain reaction (qRT-PCR) and immunohistochemistry (IHC) analyses were conducted to validate the microarray results.

**Results:**

In total, 146 differentially expressed (DE) lncRNAs and 374 DE mRNAs were identified. The DE mRNAs were enriched mostly in angiogenesis-related biological processes (BPs) and pathways by bioinformatics analysis. In addition, metabolism-related BPs (e.g., “glycogen biosynthetic process” and “metabolic process”) and pathways (e.g., “oxidative phosphorylation”) were identified. A lncRNA-mRNA co-expression network was constructed from 42 DE lncRNAs and 217 DE mRNAs. Twelve lncRNAs were predicted to have cis-regulated target genes. The microarray analysis results were validated by qRT-PCR using 5 randomly selected lncRNAs and 13 mRNAs. The IHC results revealed that both LOXL2 and FPK-1 exhibited higher protein expression levels in proliferating IH than in involuting IH. Moreover, inhibition of PFK-1 could suppress hemangioma-derived endothelial cell proliferation and migration, induce cell arrest, and reduce glucose uptake and lactate and ATP production.

**Conclusions:**

The findings suggest that the identified DE lncRNAs and mRNAs may be associated with the pathogenesis of IH. The data presented herein can improve our understanding of IH development and provide direction for further studies investigating the mechanism underlying IH.

## Introduction

Infantile hemangioma (IH) is the most common benign tumor in children, with a prevalence of 4–5% [1]. IH is predominantly found in female children; the female:male ratio ranges from 1.4:1 to 3:1 [2]. IH is usually absent at birth and exhibits a characteristic growth pattern with a proliferating phase lasting for 1 year after birth, with the most rapid growth at 5–8 weeks, followed by spontaneous involution lasting up to 5 years [3]. In the proliferating phase, the IH will enlarge, become more elevated and develop a rubbery consistency; in contrast, in the involuting phase, the IH will flatten and shrink from the center outward [4]. IHs are most frequently found in the head and neck, followed by the trunk and extremities. Indications for treatment include disfigurement, impaired function and/or a threat to life [5].

Both vasculogenesis and angiogenesis are widely accepted to contribute to the mechanism of IH development [6]. Several signaling pathways that regulate vasculogenesis and angiogenesis have been demonstrated to be associated with the pathogenesis of IH [7, 8] primarily including the vascular endothelial growth factor (VEGF) and VEGF receptor pathways, notch pathway, mammalian target of rapamycin pathway, β-adrenergic signaling pathway and angiopoietin and Tie2 signaling pathway [7] [8],. However, the exact pathogenesis of IH has not been fully elucidated.

Long noncoding RNAs (lncRNAs) are most commonly defined as transcripts longer than 200 nucleotides without protein-coding function [9]. LncRNAs were previously considered transcriptional “noise”. However, accumulated studies indicate that lncRNAs play a critical role in multiple biological processes (BPs), including the regulation of gene expression at the chromatin modification level, transcriptional and posttranscriptional processing, and cell differentiation and development [9, 10]. Recently, lncRNAs have attracted increasing attention in tumorigenesis for their important roles in stemness acquisition and maintenance, growth and metastasis of cancer cells, as well as in vascular diseases [11]. Moreover, lncRNAs have been demonstrated to play a critical role in endothelial cell (EC) differentiation and angiogenesis [12].

Several studies have reported that lncRNAs are implicated in IH [13–15]. However, the association between IH and the expression levels and biological functions of lncRNAs remains unclear. This study aimed to analyze the expression profile of lncRNAs and mRNAs in proliferating and involuting IH by microarray analysis. Additionally, using Gene Ontology (GO) and Kyoto Encyclopedia of Genes and Genomes (KEGG) pathway analysis, we identified the clinical significance of differentially expressed (DE) lncRNAs and mRNAs and validated their expression using quantitative real-time polymerase chain reaction (qRT-PCR) and immunohistochemistry (IHC) staining.

## Materials and methods

### Samples

This study was approved by the Institutional Review Board of the West China Hospital of Sichuan University. Written informed consent was obtained from the parents of all patients. All IH tissues were obtained surgically at the West China Hospital of Sichuan University. In total, tissues from six proliferating IHs and four involuting IHs were collected, and all specimens were stored at − 80 °C after excision. Detailed information on the ten patients is presented in Additional file 1: Table S1.

### Cell culture

Hemangioma-derived endothelial cell (HemEC) isolation was performed as described previously [16–18]. The HemECs were cultured with Endothelial basal medium (EBM-2, Lonza, Walkersville, MD, USA) containing 10% fetal bovine serum (Gibco), SingleQuot (Lonza), penicillin (Gibco) and streptomycin (Gibco). The cells were grown in a humidified atmosphere containing 5% CO_2_ in the air at 37 °C.

### RNA extraction

Total RNA was extracted from frozen tissues using the TRIzol reagent (Invitrogen, Carlsbad, CA, USA) and was further purified with a miRNeasy Mini Kit (QIAGEN, Valencia, CA, USA) according to the manufacturer’s instructions. RNA quantity and quality were measured with a NanoDrop 2000 spectrophotometer (Thermo Scientific, Wilmington, DE, USA), and RNA purity and integrity were assessed by agarose gel electrophoresis. Then, RNA samples were stored at -80 °C until further use.

### Microarray analysis

LncRNA and mRNA expression profiling were performed using the Affymetrix GeneChip Human Transcriptome Array 2.0 platform (Affymetrix, Inc., Santa Clara, CA, USA). This high-resolution array contains more than 6 million distinct probes, including over 245,000 mRNA and 40,000 lncRNA probes. Raw microarray data were extracted by using the Agilent Feature Extraction (v. 10.7), summarized, normalized and quality-controlled using the GeneSpring GX program software (v. 12.6.1) package (Agilent Technologies) and R program. Then microarray analysis was performed via the Gene-Cloud of Biotechnology Information (GCBI, Shanghai, China; https://www.gcbi.com.cn) platform. Briefly, the DE lncRNAs and mRNAs between the proliferating group and the involuting group were first identified. Second, GO enrichment and KEGG pathway analyses were performed with the DE genes. Finally, the DE lncRNAs and mRNAs were used to construct a lncRNA-mRNA co-expression network. LncRNAs and mRNAs significantly DE between the two groups were identified by filtering with an adjusted *P* value < 0.05 and fold change > 1.5 using SAM analysis. A volcano plot was generated to distinguish the significantly DE lncRNAs and mRNAs. Hierarchical clustering was applied to display the expression profiles of significantly DE lncRNAs and mRNAs between the two groups.

### GO and KEGG pathway analyses

GO analysis was used to categorize and describe the biological functions of the significantly DE genes using the cellular component (CC), molecular function (MF) and BP categories. Based on the KEGG database, pathway analysis was used to predict the main pathways enriched by the DE genes. Differentially expressed mRNAs were selected and uploaded into the Database for Annotation, Visualization and Integrated Discovery (DAVID, https://david.ncifcrf.gov/) for annotation and functional analysis, including gene set enrichment analysis and mapping gene sets to the KEGG pathway [19, 20]. Both GO enrichment analysis (P < 0.05 and FDR < 0.05) and KEGG pathway analysis (P < 0.05 and FDR < 0.05) of DE mRNAs were performed using the GCBI platform.

### Protein-protein interaction (PPI) network analysis

PPI networks are an important tool for the system-level understanding of cellular processes [21]. To identify hub DE mRNAs related to the development of IH, we performed PPI network analysis automatically via STRING version 11.0 online software (https://string-db.org/) [22]. Then, the PPI network was drawn with the Cytoscape software 3.6.1 (The Cytoscape Consortium, San Diego, CA, USA). Moreover, the top 10 hub genes were selected for further functional analysis using Metascape (http://metascape.org) [23].

### LncRNA-mRNA co-expression network

Based on the correlation analysis between the significantly DE lncRNAs and mRNAs, a lncRNA-mRNA co-expression network was generated to associate the lncRNAs with their potential target mRNAs using Pearson correlation. Pearson correlation coefficients > 0.97 or < − 0.97 with P-values < 0.05 were used as the standard threshold values for identifying the lncRNAs and protein-coding mRNAs. The co-expression network was first constructed via the GCBI platform and was then drawn with the Cytoscape 3.6.1 software.

### Prediction of cis-regulated targets

The prediction of cis-regulated target genes was performed based on the results of the lncRNA-mRNA co-expression analysis. mRNAs were considered cis-regulated target genes when the Pearson correlation coefficient was > 0.97 or < − 0.97 and the mRNA locus was within 10 kb of each given lncRNA.

### qRT-PCR validation

qRT-PCR analysis was performed using SYBR qPCR Master Mix (Vazyme, Nanjing, China) according to the manufacturer’s protocol. The qRT-PCR conditions were as follows: 95 °C for 30 s, followed by 40 cycles at 95 °C for 15 s and 60 °C for 30 s. All experiments were performed in triplicate. The expression levels of lncRNAs and mRNAs were quantified using the 2^−ΔΔCt^ method and normalized to GAPDH expression. All the primers used in this study are listed in Additional file 2: Table S2.

### IHC staining

To assess the protein expression and cellular localization of LOXL2 and PFK-1, fifty pairs of proliferating and involuting IH tissues were selected for IHC analysis. The 5 μm tissue sections were cut and deparaffinized by heating to 60 °C for 1 h, washed three times with xylene for 15 min each, and rehydrated by consecutive washes in 100%, 95%, and 70% ethanol followed by a 5-min wash in water. Then, sections were incubated with 3% hydrogen peroxide for 30 min to inhibit endogenous peroxidase activity and subsequently underwent consecutive washes with PBS. Antigen retrieval was carried out by heating sections twice for 20 min each in EDTA antigen retrieval buffer. Sections were blocked for 30 min in 5% serum at room temperature. Primary antibodies against LOXL2 (1:100, GTX105085, GeneTex) and PFK-1 (1:50, sc-377346, Santa Cruz) were added for incubation overnight at 4 °C. Slides were washed and incubated with secondary antibody at room temperature for 30 min. Images were acquired using a Leica microscope camera (Leica Microsystems, Wetzlar, Germany).

### Western blot analysis

Briefly, the protein concentration was determined using the Bradford protein assay kit (Bio-Rad). Then, the protein samples were separated by sodium dodecy1 sulphate–polyacrylamide gel electrophoresis, followed by electrophoretic transfer onto a nitrocellulose membrane. The membrane was incubated with primary antibody in TBST at 4 °C overnight. Next, the membrane was washed three times and incubated with the appropriate secondary antibody. The protein bands were visualized using enhanced ECL-associated fluorography.

### Lentiviral vector construction and transfection

Briefly, two specific shRNAs for PFK-1 (shRNA-1 and shRNA-2) were purchased form GeneChem Co., Ltd. (Shanghai, China) and Lipofectamine 2000 (Invitrogen, USA) was applied for transfection according to the manufacturer’s protocol. After transfection with 48 h, the cells were collected for various experiments.

### CCK-8 assay

Briefly, 5,000 transfected cells were seeded into 96-well plates for 24 h. Then, 10 μl per well of CCK-8 kit reagents were added and incubated for 2 h at 37 °C. Finally, the absorbance of each well was read at 450 nm on a microplate reader. All experiments were independently repeated at least three times.

### Migration assay

The transfected cells were plated in serum-free medium in the top chamber. The membrane without a coat (24-well insert; 8 μm pore size; Millipore, USA) and medium supplemented with 10% serum was in the lower chamber. After 24 h, the bottom of the chamber insert was stained with methanol and 0.1% crystal violet and then imaged. Each migration assay was conducted at least 3 replicates.

### Flow cytometry cell cycle analysis

The cell cycle detection kit purchased from 4A Biotech Co., Ltd. (Beijing, China) was used to detect the cell cycle. HemECs (4.0 × 10^5^/well) were plated into 6-well plates and cultured for 24 h. Then, the cells were collected by trypsinization and washed with cold PBS. Subsequently, 95% cold ethanol was used to immobilize the cells at 4 °C overnight. After washing again with PBS, the cells were incubated with RNase and then labelled with propidium iodide (PI) according to the manufacturer’s protocol. A CytoFLEX flow cytometer (Becton–Dickinson, USA) was used to detect the cell cycle. The cell cycle distribution was analyzed with the ModFIT software (BD Biosciences).

### Glucose uptake assay

The glucose uptake of HemECs was estimated using the Glucose Uptake Cell-Based Assay Kit (Cayman Chemical, USA). Briefly, HemECs (5 × 10^4^/well) were seeded in 96-well plates and then incubated at 37 °C overnight. On the next day, the cells were treated for 1 h with glucose-free medium and fluorescent 2-NBDG at a concentration in the glucose-free medium. The plate was centrifuged at room temperature for five minutes at 400 g followed by aspirating the supernatant. Then, 200 μl of Cell-Based Assay Buffer was added to each well, and the cells were analyzed immediately using a CytoFLEX flow cytometer (Becton–Dickinson, USA).

### Lactate production

Lactate levels was measured using the L-Lactate Assay Kit (Cayman Chemical, USA) according to the manufacturer’s protocol. Briefly, 1 × 10^4^ cells/well in 120 μl of culture medium were seeded in a 96-well plate for 24 h. Then, 20 µl of medium was collected into a new 96 well plate for colorimetric detection at 490 nm with a microplate reader. All experiments were performed in triplicate.

### ATP production

ATP levels was measured using an ATP Colorimetric/Fluorometric Assay kit (BioVision, USA) according to the manufacturer’s instructions. Briefly, lysates of 1 × 10^6^ cells were collected to detect the ATP concentrations using a microplate reader at 490 nm.

### Statistical analysis

Data were analyzed using SPSS version 23.0 (SPSS, Chicago, IL, USA). Continuous variables are presented as the means ± standard deviations, and the Student’s t-test was used to assess the differences between two groups. Correlation analysis was utilized for the expression levels of lncRNAs and mRNAs. P values < 0.05 were considered statistically significant.

## Results

### LncRNA and mRNA expression profiles in IH

**Fig. 1:**
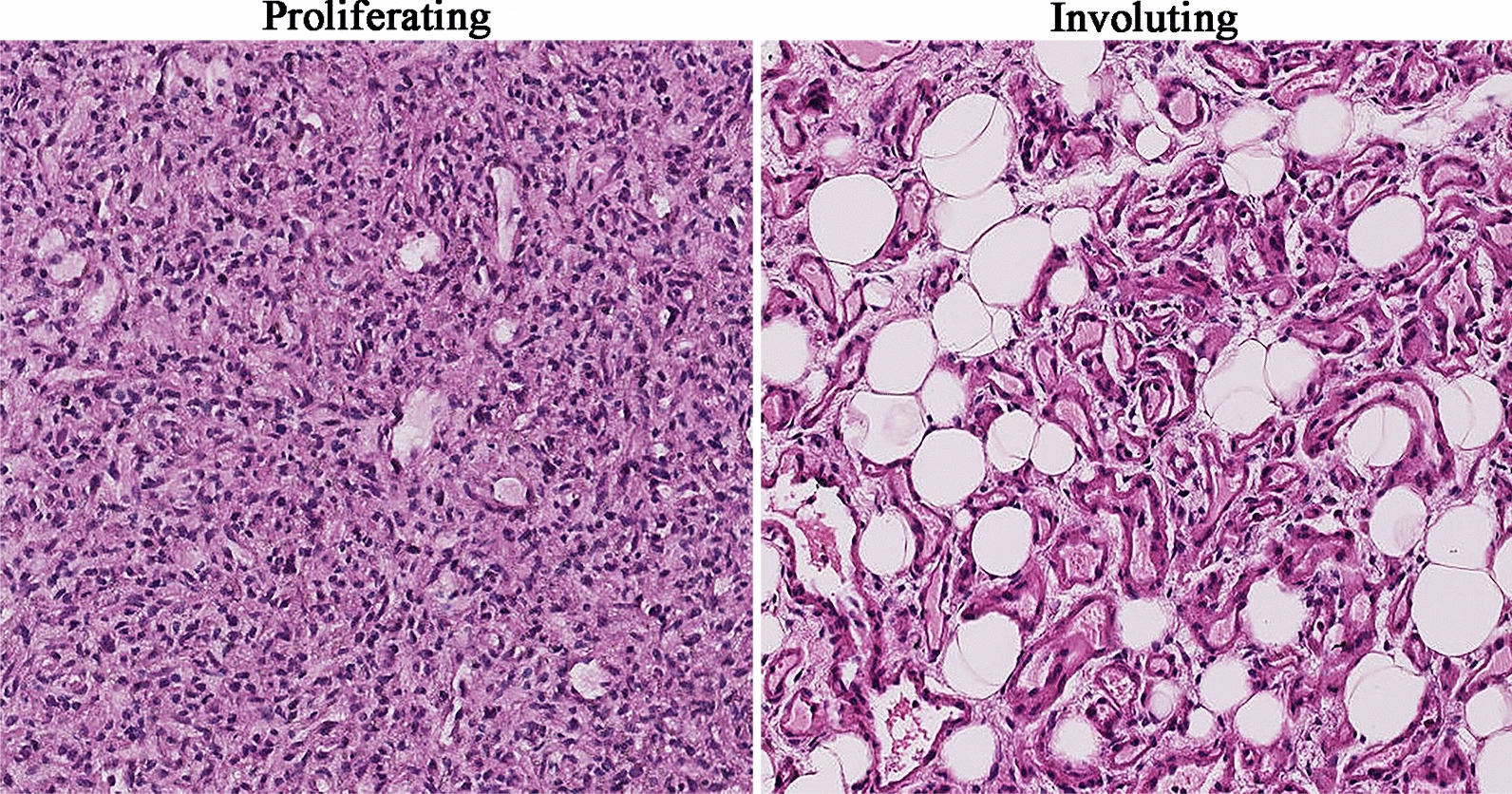
Hematoxylin-eosin-stained infantile hemangioma histopathological specimens (original magnification: × 100). The proliferating phase is characterized by densely packed tumor cells that form immature vessels. In the involuting phase, the disorganized vasculature consists of a flat endothelium and pericytes

**Fig. 2:**
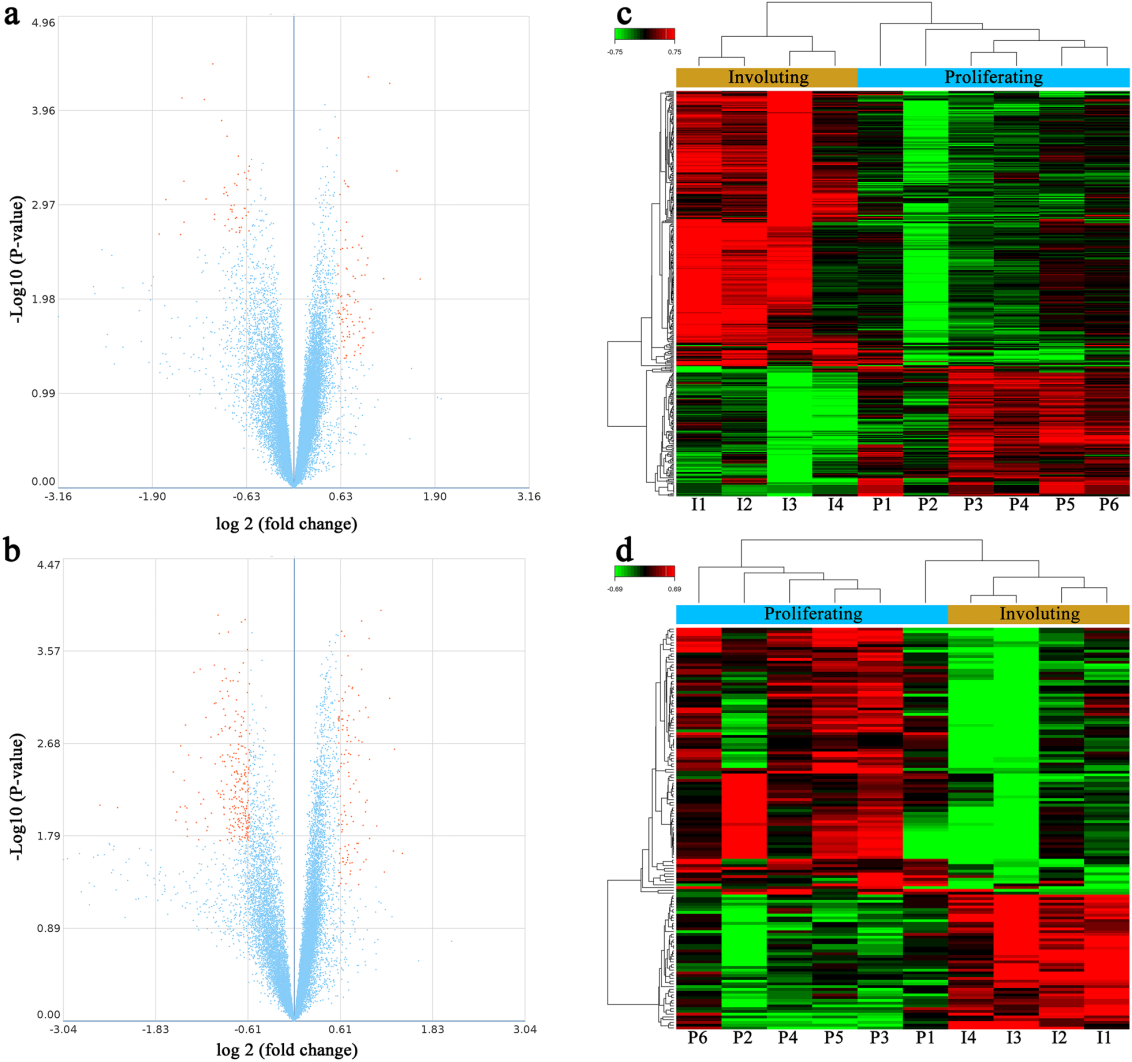
LncRNA and mRNA expression profiles in proliferating and involuting IH. Volcano plots of DE lncRNAs (**a**) and mRNAs (**b**) between the proliferating and involuting groups. The left and right orange dots indicate DE lncRNAs or mRNAs in the plot that were downregulated and upregulated, respectively. Hierarchical clustering of DE lncRNAs (**c**) and mRNAs (**d**) between the proliferating and involuting groups. Red and green indicate high relative expression and low relative expression, respectively. P1-P6: Proliferating IH tissues; I1-I4: Involuting IH tissues

To explore the expression level profiles of lncRNAs and mRNAs in IH, lncRNA and mRNA microarray analyses were performed with six proliferating IH samples and four involuting IH samples (Fig. 1). A volcano plot was generated to provide an overview of the DE lncRNAs and mRNAs in our microarray data (Fig. 2a, b). In total, 146 DE lncRNAs—97 upregulated lncRNAs and 49 downregulated lncRNAs—were identified (Additional file 3: Table S3). Of the 374 DE mRNAs, 115 were upregulated and 259 were downregulated (Additional file 4: Table S4). Hierarchical clustering analyses were used to display the lncRNA and mRNA expression patterns (Fig. 2c, d). These data implied that lncRNA and mRNA expression levels differed between proliferating and involuting IHs. The top 10 DE lncRNAs and mRNAs are shown in Tables 1 and 2, respectively.

**Table 1:** Top 10 differentially expressed long noncoding RNAs

LncRNA ID	Database source	Fold change	P-value	Feature	Rank
TCONS_00000305-XLOC_000397	Rinn lincRNA	− 2.123991	3.50E − 05	Down	1
n334063	NONCODE	2.001631	4.80E − 05	Up	2
n333116	NONCODE	2.445146	5.60E − 05	Up	3
n341453	NONCODE	− 2.830943	8.00E − 05	Down	4
n333411	NONCODE	− 2.298218	8.30E − 05	Down	5
n410169	NONCODE	− 1.956713	0.000138	Down	6
n342353	NONCODE	− 1.860167	0.000202	Down	7
n335587	NONCODE	1.516683	0.00021	Up	8
n335269	NONCODE	− 1.674877	0.000327	Down	9
TCONS_l2_00001442-XLOC_l2_001047	Broad TUCP	− 1.511823	0.000417	Down	10

**Table 2:** Top 10 differentially expressed mRNAs

Gene ID	Gene description	Fold change	P-value	Feature	Rank
NOTCH3	Notch 3	2.211008	0.000108	Up	1
LYPLAL1	Lysophospholipase like 1	− 2.00366	0.00012	Down	2
PFK1	Phosphofructokinase 1	1.56533	0.000133	Up	3
LOXL2	Lysyl oxidase like 2	1.852747	0.000137	Up	4
SNCA	Synuclein alpha	− 1.61744	0.00014	Down	5
DOCK6	Dedicator of cytokinesis 6	1.544228	0.000172	Up	6
UGP2	UDP-glucose pyrophosphorylase 2	− 1.82434	0.000175	Down	7
HSDL2	Hydroxysteroid dehydrogenase like 2	− 1.98112	0.000181	Down	8
COL18A1	Collagen type XVIII alpha 1 chain	1.582594	0.000192	Up	9
PDGFRB	Platelet derived growth factor receptor beta	1.982871	0.000202	Up	10

### GO and KEGG pathway analysis of DE mRNAs

**Fig. 3:**
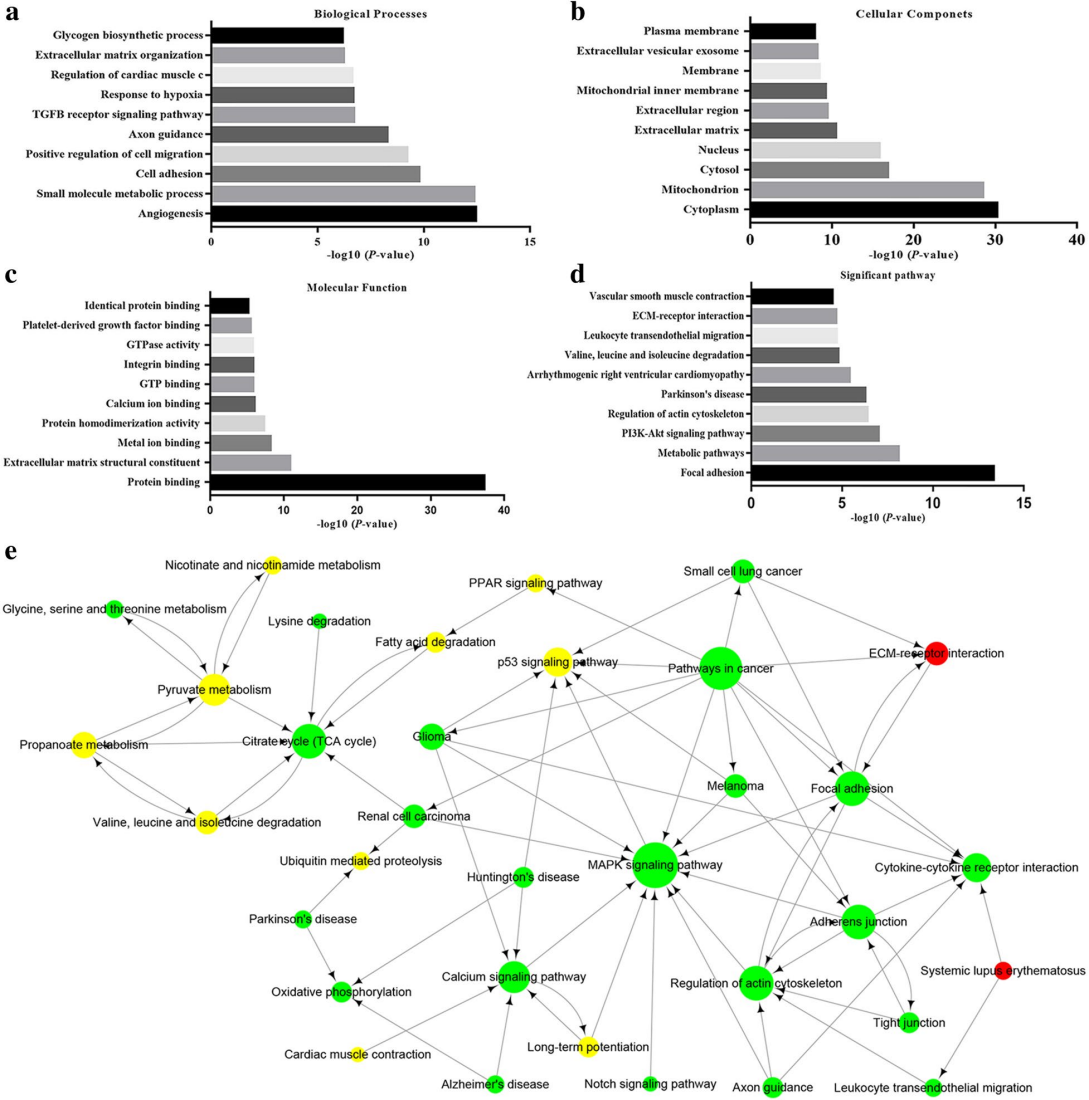
GO and KEGG pathway analyses of DE mRNAs. The top 10 most highly enriched BPs (**a**), CCs (**b**), and MFs (**c**) and most significant pathways (**d**). Pathway interaction network (**e**). Red indicates upregulated pathways, yellow indicates downregulated pathways, green indicates pathways with both upregulation and downregulation, and the node size represents the degree

Analysis of the DE mRNAs via GO and KEGG pathway analysis could provide a clue to the role of DE mRNAs in the IH disease process. All DE mRNAs were used in GO analysis, and the top 10 GO terms, including those in the BP, CC and MF categories, are shown in Fig. 3a–c. A total of 139 BPs were identified (Additional file 5: Table S5). The most significantly enriched GO term was “angiogenesis”, followed by “small molecule metabolic process”, “cell adhesion” and “positive regulation of cell migration”. Moreover, in addition to classical BPs associated with cell proliferation and migration in IH, interesting metabolism-related BPs were also found, including “glycogen biosynthetic process”, “branched-chain amino acid catabolic process” and “mitochondrial electron transport, NADH to ubiquinone”. In total, 19 (13.7%) metabolic BPs were identified and are shown in Table 3.

**Table 3:** Nineteen metabolic biological processes associated with infantile haemangioma

GO ID	GO Term	Count	P-value	FDR	**Rank**
GO:0044281	Small molecule metabolic process	43	4.51E − 13	3.42E − 10	1
GO:0005978	Glycogen biosynthetic process	5	6.86E − 07	0.000104	2
GO:0009083	Branched-chain amino acid catabolic process	4	2.89E − 05	0.002057	3
GO:0006120	Mitochondrial electron transport, NADH to ubiquinone	5	3.49E − 05	0.00227	4
GO:0006511	Ubiquitin-dependent protein catabolic process	9	4.11E − 05	0.002355	5
GO:0006979	Response to oxidative stress	7	5.35E − 05	0.002899	6
GO:0030574	Collagen catabolic process	6	7.17E − 05	0.003509	7
GO:0044255	Cellular lipid metabolic process	8	8.65E − 05	0.003858	8
GO:0005977	Glycogen metabolic process	4	0.000114	0.004677	9
GO:0022904	Respiratory electron transport chain	6	0.000195	0.006581	10
GO:0007005	Mitochondrion organization	4	0.00031	0.009577	11
GO:0034641	Cellular nitrogen compound metabolic process	8	0.000409	0.010748	12
GO:0032981	Mitochondrial respiratory chain complex I assembly	3	0.000634	0.014786	13
GO:0006184	GTP catabolic process	6	0.000866	0.017987	14
GO:0006108	Malate metabolic process	2	0.00296	0.039357	15
GO:0009404	Toxin metabolic process	2	0.00296	0.039357	16
GO:0072593	Reactive oxygen species metabolic process	3	0.003438	0.043799	17
GO:0006554	Lysine catabolic process	2	0.003924	0.045409	18
GO:0055114	Oxidation–reduction process	5	0.004046	0.04612	19

KEGG pathway analysis identified 64 pathways (Additional file 6: Table S6). The DE mRNAs were mostly enriched in the “focal adhesion”, “metabolic pathways” and “PI3K-Akt signaling pathway” KEGG pathways. The top 10 significant pathways are shown in Fig. 3d. In addition, 11 (17.2%) metabolism-related pathways were found and are shown in Table 4. Then, we conducted pathway network analysis using all the significant pathways to illustrate the critical pathways in the process of hemangioma neovascularization (Fig. 3e). The pathway network comprised 34 network nodes and 74 edges. In the network, the “MAPK pathway”, “pathway in cancer” and “Citrate cycle (TCA cycle)” KEGG pathways were considered the most relevant pathways because they had the highest degree values, indicating that these pathways may play the most important role in IH.

**Table 4:** Ten metabolism-related pathways associated with infantile hemangioma

Pathway ID	Pathway Name	Count	P-value	FDR	Rank
1100	Metabolic pathways	33	7.93E − 09	7.22E − 07	1
280	Valine, leucine and isoleucine degradation	6	3.96E − 06	0.00012	2
190	Oxidative phosphorylation	7	0.000307	0.003189	3
620	Pyruvate metabolism	4	0.000821	0.005745	4
71	Fatty acid degradation	4	0.001078	0.007008	5
2010	ABC transporters	4	0.001078	0.007008	6
20	Citrate cycle (TCA cycle)	3	0.004207	0.020696	7
640	Propanoate metabolism	3	0.005076	0.023095	8
260	Glycine, serine and threonine metabolism	3	0.007706	0.031874	9
310	Lysine degradation	3	0.01695	0.058205	10

### PPI network analysis

**Fig. 4:**
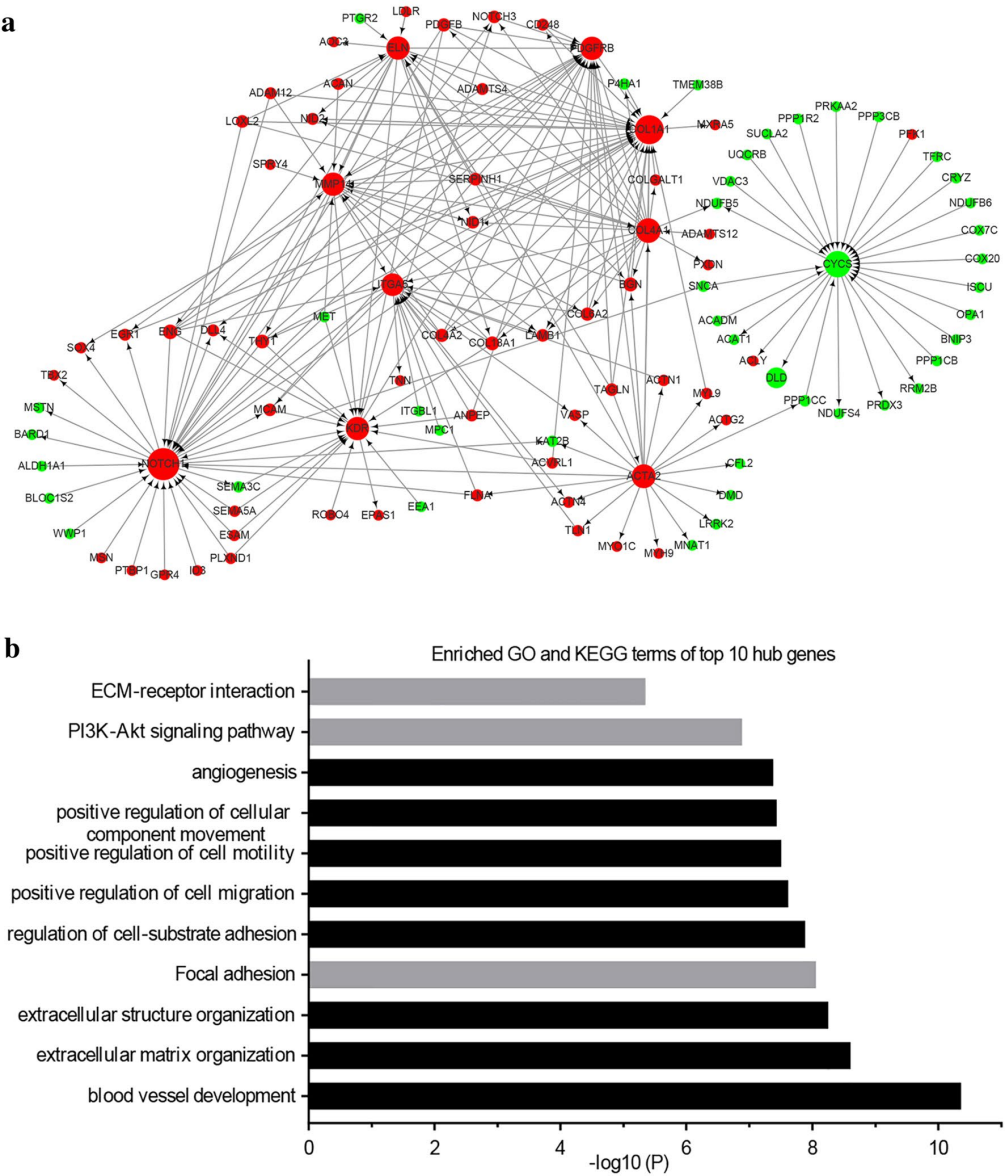
The top 10 hub genes in the protein–protein interaction network (**a**). Red and green indicate upregulated and downregulated mRNAs, respectively. The node size represents the degree. GO and KEGG terms enriched in the top 10 hub genes (**b**). Black and gray indicate BPs and pathways, respectively

Genes mutually affect the expression of each other. To investigate the functions of DE genes at the protein level and determine the core genes in IH, protein–protein network analysis was performed using STRING. The resulting PPI network contained 374 nodes and 935 edges (Additional file 7: Fig. S1). After degree calculation, 31 hub genes were identified with a degree ≥ 15 (Table 5). Among these hub genes, NOTCH1, COL1A1, CYCS, COL4A1, ACTA2, PDGFRB, KDR, MMP14, ELN and ITGA5 were the 10 hub genes with the closest connections to other nodes (Fig. 4a). These top 10 genes were most highly enriched in multiple BPs (including “extracellular matrix organization” and “angiogenesis”) and 2 KEGG pathways (“focal adhesion” and “PI3K/Akt signaling pathway”), as shown by Metascape analysis (Fig. 4b).

**Table 5:** Thirty-one hub genes with a degree of ≥ 15 in the protein–protein interaction network

Gene ID	Fold Change	P-value	Feature	Out Degree	In Degree	Total Degree	Rank
NOTCH1	1.505646	0.000815	Up	13	22	35	1
COL1A1	1.530545	0.047257	Up	20	10	30	2
CYCS	− 1.645191	0.007507	Down	7	19	26	3
COL4A1	2.501585	0.002376	Up	19	4	23	4
ACTA2	1.609589	0.029662	Up	21	1	22	5
PDGFRB	1.982871	0.000202	Up	3	18	21	6
KDR	2.478637	0.022919	Up	4	17	21	7
MMP14	1.646649	0.001126	Up	9	12	21	8
ELN	1.559719	0.029079	Up	11	10	21	9
ITGA5	1.859768	0.002024	Up	8	12	20	10
PPP1CC	− 1.611769	0.005672	Down	6	13	19	11
UBE2D1	− 2.344914	0.002874	Down	13	6	19	12
PPP1CB	− 1.590005	0.005132	Down	16	3	19	13
BGN	1.619226	0.012656	Up	8	10	18	14
COL4A2	2.395693	0.000766	Up	16	2	18	15
DLD	− 1.877071	0.001431	Down	0	17	17	16
TLN1	1.548241	0.001011	Up	5	12	17	17
COL6A2	1.539987	0.002659	Up	8	9	17	18
FLNA	1.699094	0.000618	Up	12	5	17	19
ACTN1	1.506622	0.012742	Up	13	4	17	20
ENG	1.567089	0.00081	Up	15	2	17	21
THY1	1.920826	0.007751	Up	6	10	16	22
COL18A1	1.582594	0.000192	Up	11	5	16	23
CUL2	− 1.5414	0.013956	Down	16	0	16	24
NDUFB5	− 1.639639	0.004704	Down	2	13	15	25
MYL9	1.622037	0.02276	Up	3	12	15	26
UBE2B	− 1.61738	0.001153	Down	4	11	15	27
LRRK2	− 1.80695	0.001503	Down	7	8	15	28
CUL5	− 1.900919	0.007236	Down	10	5	15	29
ACADM	− 1.721258	0.003933	Down	11	4	15	30
ACTG2	1.564071	0.026988	Up	13	2	15	31

### Construction of the lncRNA-mRNA co-expression network

**Fig. 5:**
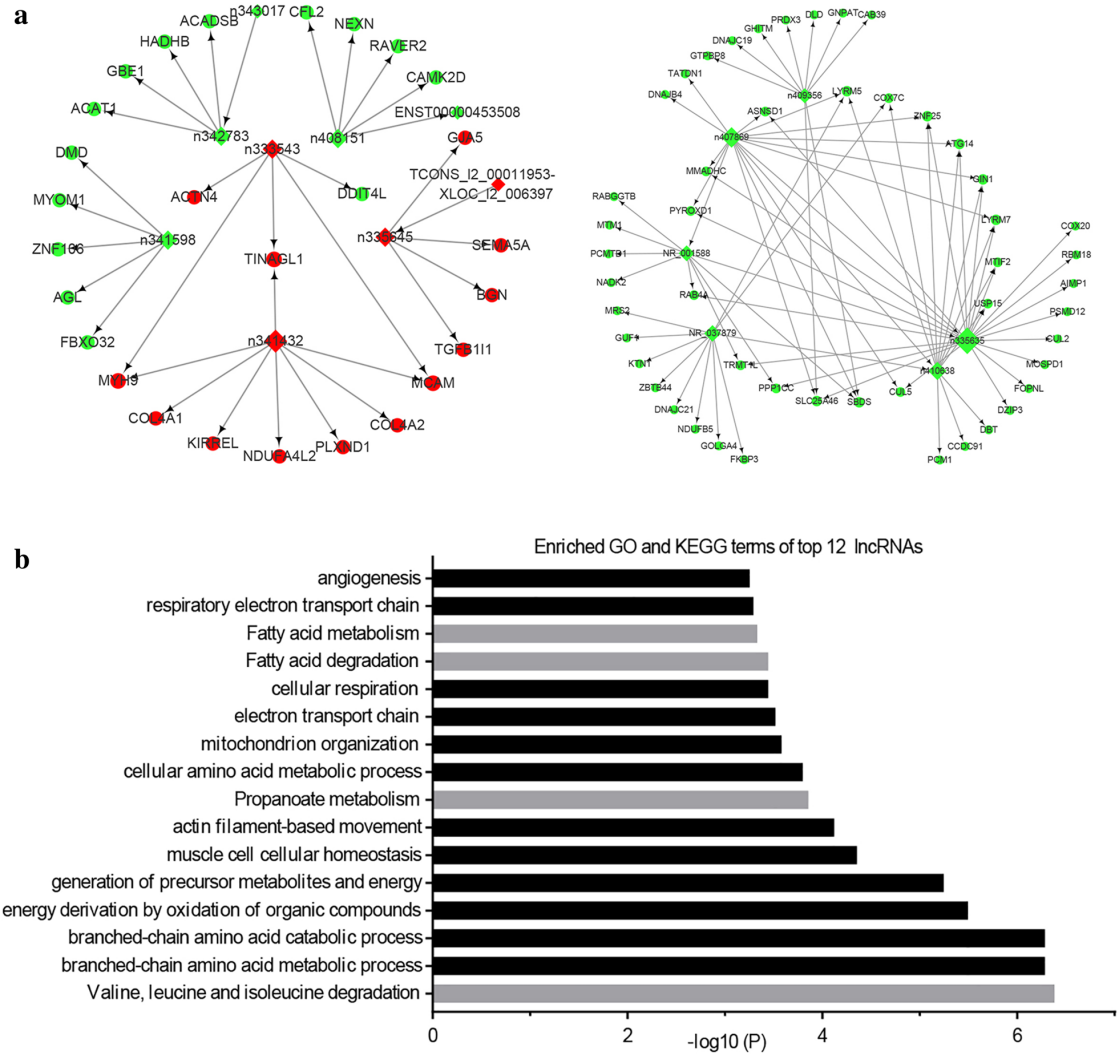
LncRNA-mRNA co-expression network. The circles and diamonds represent mRNAs and lncRNAs, respectively. Red and green indicate upregulation and downregulation, respectively. The node size represents the degree

**Fig. 6:**
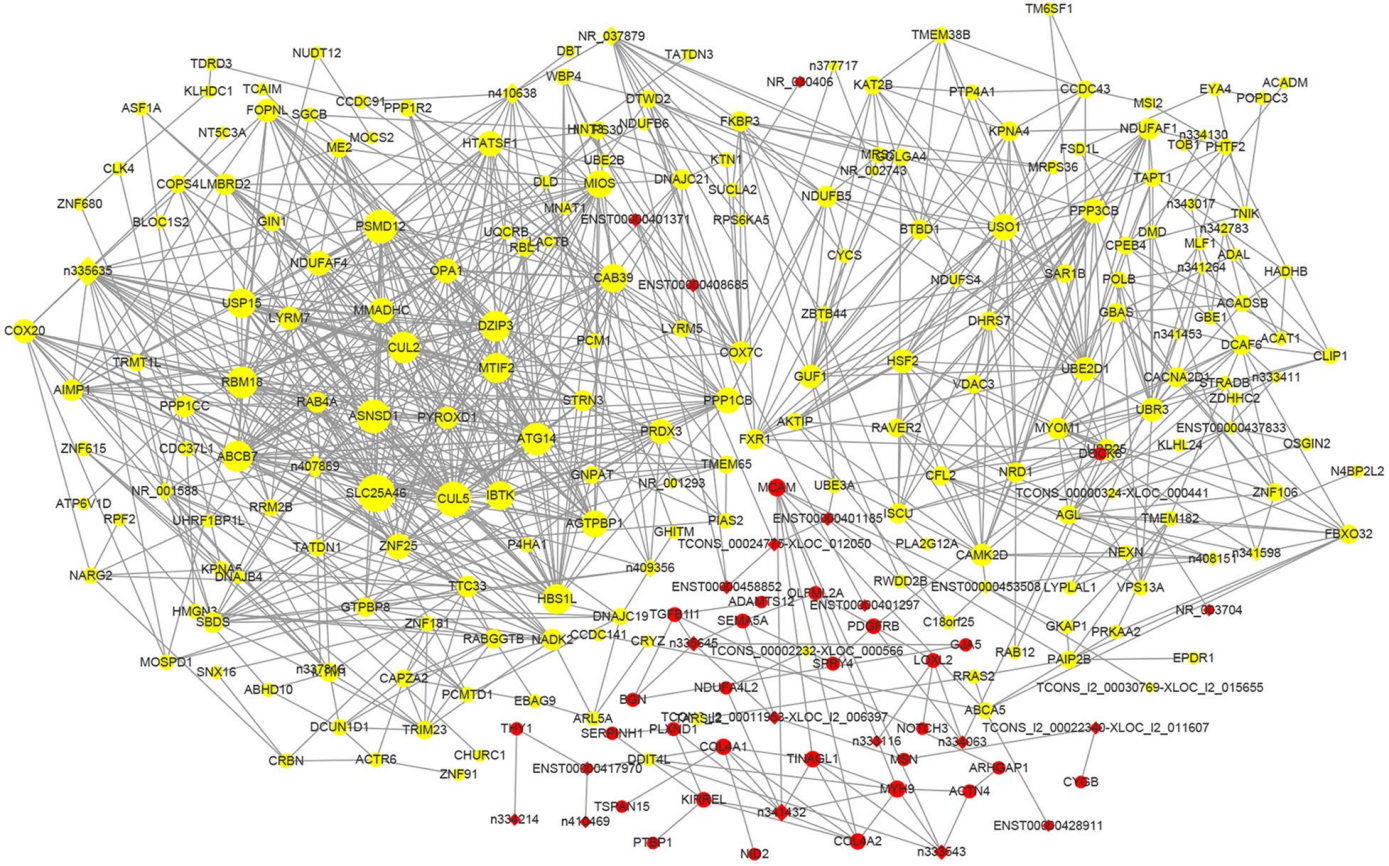
Co-expression network of twelve lncRNAs with their potential target mRNAs (Fig. 5a). The circles and diamonds represent mRNAs and lncRNAs, respectively. Red and green indicate upregulation and downregulation, respectively. The node size represents the degree. GO and KEGG terms enriched in the top 12 lncRNAs in the co-expression network (Fig. 5b). Black and gray indicate BPs and pathways, respectively

To identify hub regulatory factors associated with IH, lncRNA-mRNA co-expression network analysis was performed with 146 DE lncRNAs and 374 DE mRNAs. In total, 42 lncRNAs and 217 mRNAs were included in the co-expression network, which comprised 259 network nodes and 915 connections (Fig. 5). Among these included mRNAs and lncRNAs, 28 mRNAs and 15 lncRNAs were upregulated and 189 mRNAs and 27 lncRNAs were downregulated. In the co-expression network, many mRNAs were correlated with a single lncRNA and vice versa. We then calculated the core degree to estimate the relative significance of an individual lncRNA or mRNA in the co-expression network. The top twelve DE lncRNAs with a relatively high core degree (≥ 5) may be critical to the regulation of the pathogenetic mechanism underlying IH (Fig. 6a). However, to date, the functions of most lncRNAs have not been well-annotated. Therefore, we can predict the potential role of lncRNAs by analyzing the function of DE mRNAs via GO and KEGG pathway analyses [24]. GO and KEGG pathway analysis of the co-expressed mRNAs via Metascape indicated that the top 12 lncRNAs were mostly involved in cell metabolism-associated BPs and pathways (Fig. 6b).

### Prediction of cis-regulated targets of lncRNAs

To explore the functions of lncRNAs in IH, we predicted the cis-regulated genes of the DE lncRNAs using co-expression network analysis. Altogether, 15 lncRNAs were predicted to have cis-regulated target genes. These lncRNAs and their target genes are shown in Table 6.

**Table 6:** Twelve lncRNAs and their cis-regulated target genes

LncRNA ID	Database source	LncRNA feature	Target gene	Gene feature	Correlation
n335635	NONCODE	Down	RAB4A	Down	0.979936
n407869	NONCODE	Down	TATDN1	Down	0.976091
n409356	NONCODE	Down	DNAJC19	Down	0.984042
n341598	NONCODE	Down	FBXO32	Down	0.983656
n342783	NONCODE	Down	ACAT1	Down	0.991786
n408151	NONCODE	Down	CFL2	Down	0.994730
n341264	NONCODE	Down	CACNA2D1	Down	0.974870
n334130	NONCODE	Down	DMD	Down	0.977736
n341432	NONCODE	Up	COL4A2	Up	0.999611
n333543	NONCODE	Up	MYH9	Up	0.995991
n334063	NONCODE	Up	PDGFRB	Up	0.985527
n334214	NONCODE	Up	THY1	Up	0.993982

### Validation of differential lncRNA and mRNA expression

**Fig. 7:**
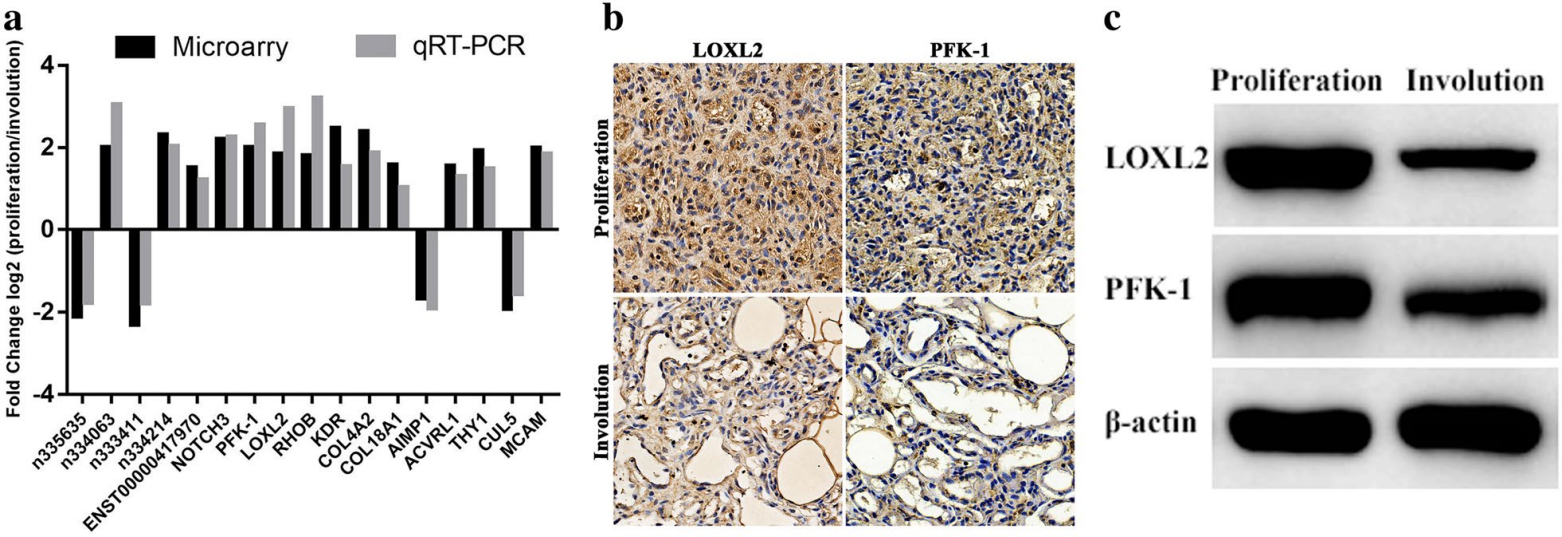
qRT-PCR validation of 5 randomly selected DE lncRNAs and 13 DE mRNAs in the microarray analysis (Fig. 6a). The heights of the columns represent the mean values of log-transformed fold changes in expression. IHC staining (**b**) and western blot (**c**) analysis of two of the most significantly DE genes—LOXL2 and PFK-1. Both LOXL2 and PFK-1 were more highly expressed in proliferating IH than in involuting IH (original magnification: × 400)

To validate our microarray results, we randomly selected 5 lncRNAs (3 upregulated and 2 downregulated) and 12 mRNAs (10 upregulated and 2 downregulated) and measured their expression levels via qRT-PCR. The qRT-PCR results showed that the expression of lncRNAs n334063, n334214 and ENST00000417970 was upregulated, whereas that of lncRNAs n333411 and n335635 was downregulated. In addition, two mRNAs, AIMP1 and CUL5, were downregulated, and the remaining mRNAs, including NOTCH3, PFK-1, LOXL2, RHOB, KDR, COL4A2, COL18A1, ACVRL1, THY1 and MCAM, were upregulated. The qRT-PCR results were thus consistent with the microarray analysis results (Fig. 7a). Moreover, we further selected two of the most significantly DE mRNAs, in which we were the most interested, and assessed their protein expression levels by IHC analysis and western blot (Fig. 7b, c). The IHC staining and western blot results showed that both LOXL2 and FPK-1 exhibited higher protein expression in proliferating IH than in involuting IH. Moreover, both LOXL2 and FPK-1 were located mainly in the cytoplasm (Fig. 7b).

### Suppression of PFK-1 inhibits HemEC growth and migration and induces cell cycle arrest

**Fig. 8:**
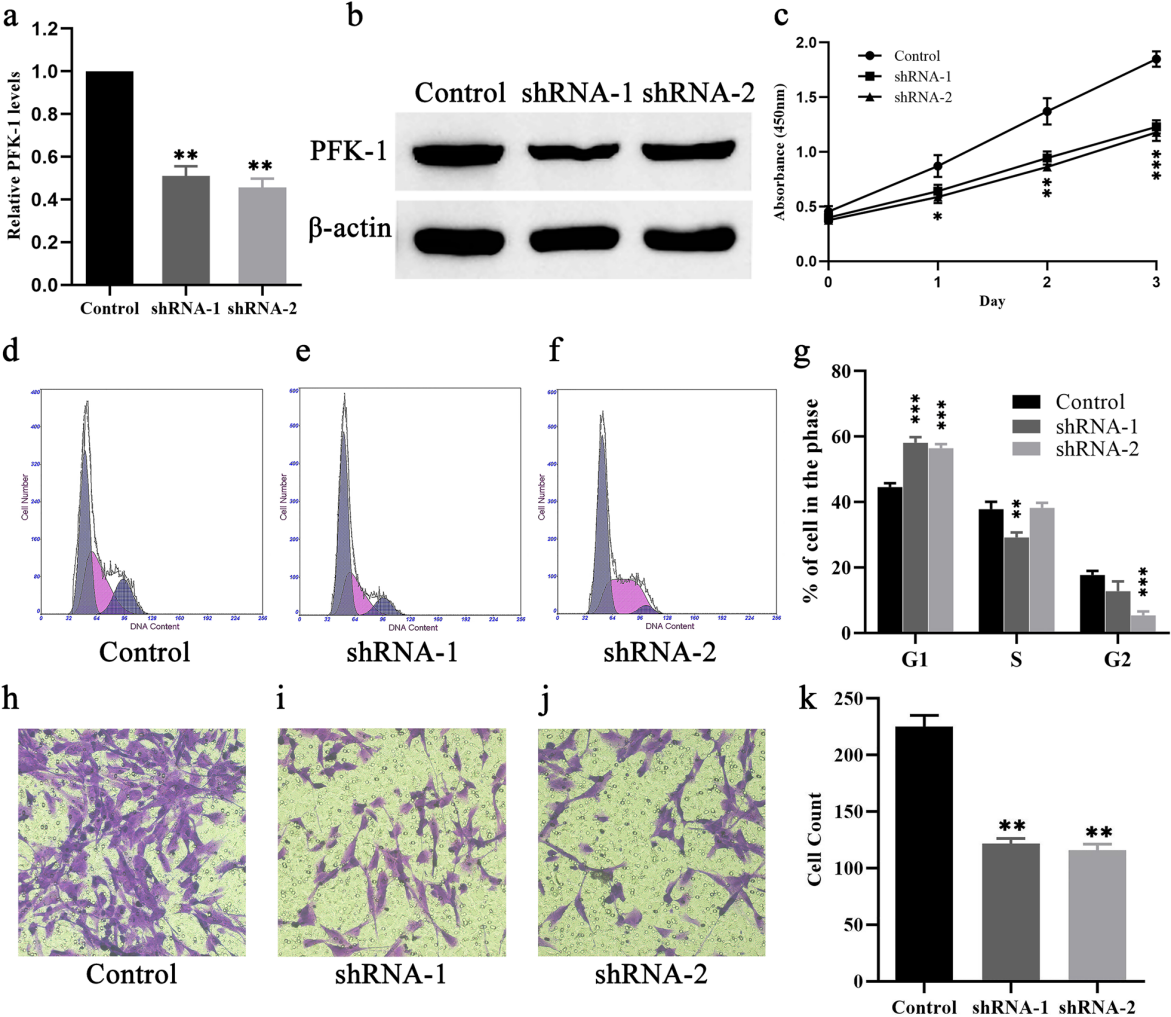
Suppression of PFK-1 inhibits HemEC growth and migration and induces cell cycle arrest. **a** Quantitative PCR and **b** western blot analysis was used to confirm the suppression rate of PFK-1 with shRNAs in HemECs. **c** CCK-8 assay was used to determine the effects of shPFK-1 on HemEC proliferation. (**d–f**) Inhibition of PFKFB3 with shRNA induces HemEC cell cycle arrest in the G1 phase. (**g–i**) Transwell assays were performed to measure the effects of PFK-1 on HemEC migration (Magnification × 100). *P < 0.05, **P < 0.01, ***P < 0.001

To investigate the biological roles of PFKFB3 in the progression of HemECs, we constructed lentiviral vectors expressing shRNAs targeting PFK-1, and then infected the HemECs with these two shRNAs lentivirus (shPFK-1). The mRNA and protein expression levels of PFK-1 were significantly inhibited by transfection with shRNAs (Fig. 8a, b). Then, a CCK-8 assay was performed to assess the effects of PFK-1 on HemEC proliferation. Compared with the negative control group, cell proliferation was significantly decreased in HemECs transfected with shPFK-1 (Fig. 8c). Furthermore, a cell cycle assay was performed to confirm the effect of PFK-1 on cell proliferation by flow cytometry. As shown in Fig. 8d–g, cell cycle was arrested in the G1 phase in HemECs transfected with the shPFK-1 lentivirus. The effect of PFK-1 knockdown on cell migration was evaluated by a Transwell assay in vitro. HemEC migration was significantly suppressed after PFK-1 knockdown (Fig. 8h–k). The above results suggest that inhibition of PFK-1 suppresses HemEC proliferation and migration and induces cell cycle arrest.

### Suppression of PFK-1 reduces glucose uptake, lactate secretion and ATP production

**Fig. 9:**
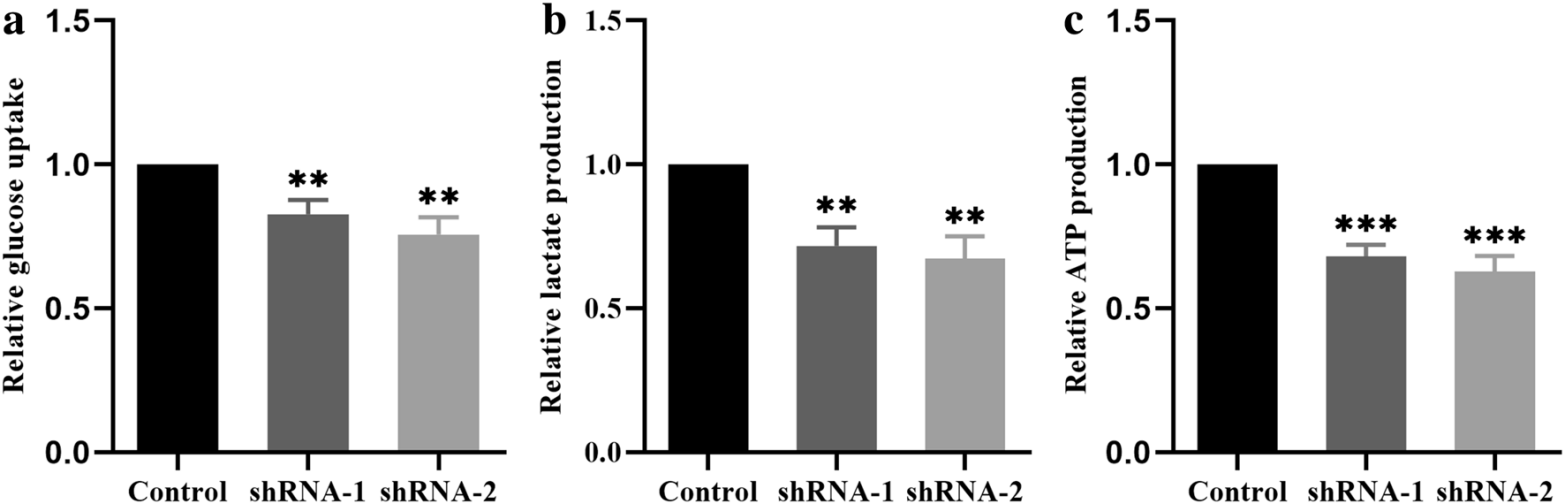
Suppression of PFK-1 reduces glucose uptake, lactate secretion and ATP production. **a** Glucose uptake, **b** lactate production and **c** ATP production were examined to investigate the effects of shPFK-1 on glycolysis flux in HemECs. The same experiments were performed in triplicate. Data are the mean ± SE. **P < 0.01, ***P < 0.001

To investigate whether PFK-1 knockdown affects glycolysis in HemECs, glucose uptake and lactate production assays were performed. As shown in Fig. 9a, b, both glucose uptake and lactate secretion were markedly decreased in PFK-1-shRNA-treated HemECs. ATP production was also significantly lower in PFK-1-shRNA-infected HemECs when compared with the control group. These results revealed that inhibition of PFK-1 could affect glycolysis through by decreasing glycolytic flux.

## Discussion

Aberrant expression of lncRNAs is involved in the pathogenesis and progression of many diseases by regulating gene expression profiles. However, the expression patterns and potential functions of lncRNAs in IH development and pathogenesis are not completely clear. Several previous studies have identified lncRNA and mRNA expression in IH and normal tissues [13, 25]. Liu and his colleague found 2116 DE lncRNAs and 2653 DE mRNAs in IH compared with adjacent normal tissues [13]. In these studies, several lncRNAs, including MEG3, Linc0152 and MALAT1, were identified to play a critical role in the regulation of tumor angiogenesis in IH [13] and demonstrated to be associated with the development of IH [14, 26–28]. However, these lncRNAs were not identified in the study by Li et al., which identified 144 DE mRNAs and 256 DE lncRNAs in IH compared with matched normal tissues [25]. To further investigate the role of lncRNAs and mRNAs in proliferating and involuting IH specimens, we analyzed the expression profile of lncRNAs and mRNAs using microarray technology. The microarray data identified 146 lncRNAs and 374 mRNAs as significantly DE between the two groups, and these findings were then validated by qRT-PCR. Although the DE lncRNAs identified in this study were not found in the previous study, many DE mRNAs including COL18A1, PDGFRB, COL4A2, THY1, ACVRL1, ANGPL1 and IFI6 were identified in earlier studies [13, 25, 29]. Collectively, these results indicated that these DE lncRNAs and mRNAs may play considerable roles in the development of IH.

To assess the biological functions enriched by the DE mRNAs, we further performed GO and KEGG pathway analyses. The GO analysis results showed that angiogenesis-related BPs, including “angiogenesis”, “cell adhesion” and “positive regulation of cell migration”, were markedly involved in the pathogenesis of IH. In addition, KEGG pathway analysis indicated that the DE mRNAs were mainly involved in angiogenesis-related pathways, including “focal adhesion”, “regulation of actin cytoskeleton” and “PI3K-Akt signaling pathway”. These findings further confirmed those of previous studies [7, 29]. Combined with the GO and KEGG pathway analysis results, our data confirmed that angiogenesis plays a critical role in the pathogenesis of IH.

The cellular components of IH include ECs, pericytes and other cells (i.e., mast cells and stem cells) [7, 8]. During the proliferating phase of IH, ECs are the predominant cells. During angiogenesis, ECs can rapidly switch from a quiescent state to an active, proliferative, migratory state in response to growth factor stimulation, primarily through VEGF [30]. The changed EC state requires vast amounts of energy to meet the bioenergetic and biomass demands of cell proliferation and migration [31]. Hence, ECs must increase their metabolic activity to quickly increase their energy generation. ECs are involved in several metabolic pathways, including the glycolysis, hexosamine biosynthesis, polyol, oxidative metabolism, amino acid metabolism, and fatty acid metabolism pathways [30, 32]. These metabolic pathways play distinct and essential roles during vessel formation. Recent studies have highlighted the importance of EC metabolism as a driving force of angiogenesis [31]. Therefore, targeting EC metabolism could offer new therapeutic opportunities to combat angiogenesis [33]. Interestingly, in the present study, some metabolism-related BPs (e.g., “glycogen biosynthetic process”, “metabolic process”, “cellular lipid metabolic process”, and “respiratory electron transport chain”) and pathways (e.g., “oxidative phosphorylation”, “pyruvate metabolism”, “citrate cycle”, and “fatty acid degradation”) were also found in IH, suggesting that cell metabolism participates and plays a vital role in the development of IH.

In tumor ECs, glycolysis is the predominant method of energy generation even in the presence of O_2_, although glycolysis is much more inefficient than oxidative phosphorylation (OXPHOS) [31]. During angiogenesis, glycolysis is further accelerated [30]. This phenomenon has several explanations, as follows: 1) it leads to environmental acidosis, which is toxic to normal cells but harmless to cancer cells; 2) it provides more carbon skeletons for biosynthesis than OXPHOS; 3) it produces ATP much faster than OXPHOS; and 4) it allows adaptation to hypoxia [34, 35]. In 2013, Bock et al. demonstrated that both human umbilical vein ECs (HUVECs) and hemangioma ECs (Hem-ECs) were highly glycolytic [36]. Moreover, this group found that glycolysis was even higher in Hem-ECs than in other types of ECs [36]. PFK-1, one of the most important rate-limiting enzymes in glycolysis, converts fructose-6-phosphate (F6P) to fructose-1,6-bisphosphate (F1,6P2). Fructose-2,6-bisphosphate (F2,6P2), synthesized by phosphofructokinase-2/fructose-2,6-bisphosphatase (PFKFB) enzymes, is an allosteric activator of PFK-1 and the most potent stimulator of glycolysis [37]. Among PFKFB isoenzymes, PFKFB3 is not only the most abundant isoenzyme in ECs [36], but also has much more efficient kinase activity than bisphosphatase activity [38]. A previous study reported that PFKFB3 can regulate EC proliferation and stimulate vessel sprouting, implying that glycolysis can regulate angiogenesis [36]. Indeed, inhibition of PFKFB3 reduced pathological angiogenesis [39], induced tumor vessel normalization, downregulated glycolytic activity in pericytes, tightened the EC barrier, impaired metastasis and improved chemotherapy [40]. In addition, PFK-1 can promote tumorigenesis by activating the AKT pathway [41]. However, the cyclin D3–cyclin-dependent kinase 6 (CDK6) complex can inhibit the catalytic activity of PFK-1 in the glycolytic pathway, suggesting a direct link between the cell cycle and cell metabolism [42]. The PI3K-Akt pathway and cyclins, with their associated kinases, CDKs, were demonstrated to participate in the development of IH in our previous study [7, 16]. Recently, glycolysis-associated molecules, including GLUT-1, HK2, PFKFB3, PKM2 and LDHA, had higher expression in HemECs compared with HUVECs [43]. In the present study, although no lncRNA was found to regulate its expression, PFK-1 was one of the most significantly DE mRNAs and was strongly expressed in proliferative IH tissues, as shown by western blot and IHC staining. Besides, we further investigated the roles of PFK-1 in cell proliferation and migration and the cell cycle. Our results revealed that silencing PFK-1 with shRNA significantly inhibited HemEC proliferation and migration and induced cell cycle arrest. In addition, we founded that PFK-1 knockdown caused a marked decrease in glucose uptake and lactate secretion in HemECs, which could affect ATP production from glycolysis by inhibiting glycolysis flux. Therefore, based on these studies, we inferred that the glycolytic activator PFK-1 also plays an important role in angiogenesis in IH. Further studies on the role of PFK-1 in the pathogenesis of IH are warranted.

LOXL2, a member of the lysyl oxidase family, was reported to have an important role in promoting angiogenesis [44]. However, inhibition of LOXL2 enhanced antiangiogenic effects in angiogenic tumors [45]. Several mechanisms explain the role of LOXL2 in angiogenesis, including activating focal adhesion kinase, increasing the expression of VEGF, promoting epithelial-mesenchymal transition, and inducing hypoxia-inducible factor-1α [46]. These studies indicate that LOXL2 has a promising role in vascularized tumors. Therefore, more studies are needed to further investigate the roles of LOXL2 in IH.

## Conclusion

This study provided a comprehensive bioinformatics analysis of lncRNA and mRNA expression profiles in proliferating and involuting IH. The identified DE lncRNAs and mRNAs may be associated with the pathogenesis of IH. Moreover, inhibition of the glycolytic activator PFK-1 could suppress HemEC proliferation and migration, induce cell arrest, and reduce glucose uptake and lactate and ATP production. The data presented here could improve our understanding of IH development and provide new directions for further studies investigating the mechanism underlying IH. Additional studies are required to further determine the exact role of DE lncRNAs and mRNAs in IH.

## Supplementary information

**Additional file 1: Table S1.** Clinical features of ten patients with infantile hemangioma.

**Additional file 2: Table S2.** Primers used for qRT-PCR analysis of lncRNAs and mRNAs.

**Additional file 3: Table S3.** Differentially expressed lncRNAs between proliferating and involuting infantile hemangioma.

**Additional file 4: Table S4.** Differentially expressed mRNAs between proliferating and involuting infantile hemangioma.

**Additional file 5: Table S5.** GO analysis of differentially expressed mRNAs.

**Additional file 6: Table S6.** KEGG pathway analysis of differentially expressed mRNAs.

**Additional file 7: Figure S1.** Protein-protein interaction network analysis by STRING.

## Data Availability

The data used to support the findings of this study are available from the corresponding author on reasonable request.

## Abbreviations

IH: Infantile hemangioma
VEGF: Vascular endothelial growth factor
LncRNAs: Long noncoding RNAs
BPs: Biological processes
EC: Endothelial cell
GO: Gene ontology
KEGG: Kyoto Encyclopaedia of Genes and Genomes
DE: Differentially expressed
qRT-PCR: Quantitative real-time polymerase chain reaction
IHC: Immunohistochemical
TCA: Citrate cycle
HUVEC: Human umbilical vein EC
CDK6: Cyclin D3–cyclin-dependent kinase 6
HemEC: Hemangioma-derived endothelial cell
PPI: Protein–protein interaction
