# Supplementary material for: Microarray expression profile of mRNAs and long noncoding RNAs and the potential role of PFK-1 in infantile hemangioma
Source: Cell Div. 2021 Jan 11;16:1. doi: 10.1186/s13008-020-00069-y (PMC7802351; doi:10.1186/s13008-020-00069-y)
Supplement: Supplementary file 1 — Additional file 1: Table S1. Clinical features of ten patients with infantile hemangioma. [file 13008_2020_69_MOESM1_ESM.docx]

**Table S1.** Clinical features of ten patients with infantile hemangioma.

| **Rank** | **Sex** | **Age** | **Location** | **Growth Phase** | **Patient No.** |
| --- | --- | --- | --- | --- | --- |
| 1 | Female | 8 months | Tempus | Proliferation | P1 |
| 2 | Female | 6 months | Thoracic wall | Proliferation | P2 |
| 3 | Female | 5 months | Elbow | Proliferation | P3 |
| 4 | Female | 6 months | Abdominal wall | Proliferation | P4 |
| 5 | Male | 11 months | Thoracic wall | Proliferation | P5 |
| 6 | Female | 6 months | Neck | Proliferation | P6 |
| 7 | Female | 29 months | Neck | Involution | I1 |
| 8 | Male | 20 months | Neck | Involution | I2 |
| 9 | Male | 13 months | Thoracic wall | Involution | I3 |
| 10 | Male | 14 months | Back | Involution | I4 |
