# Supplementary material for: Microarray expression profile of mRNAs and long noncoding RNAs and the potential role of PFK-1 in infantile hemangioma
Source: Cell Div. 2021 Jan 11;16:1. doi: 10.1186/s13008-020-00069-y (PMC7802351; doi:10.1186/s13008-020-00069-y)
Supplement: Supplementary file 2 — Additional file 2: Table S2. Primers used for qRT-PCR analysis of lncRNAs and mRNAs. [file 13008_2020_69_MOESM2_ESM.docx]

**Table S2.** Primers used for qRT-PCR analysis of lncRNAs and mRNAs.

| **LncRNA/mRNA ID** | **Forward** | **Reverse** |
| --- | --- | --- |
| n335635 | GGCTCCACAGCATGGAATC | CTGGCTATCAATGCCAGAGTC |
| n334063 | TCCATCCCTCTGTTCTCCTG | ATGGTGTCTTGCTGCTGATG |
| n333411 | GGCTCTAACGGGGCTTTCTA | ATGGAACCTGGCGTTCTG |
| n334214 | GGACTGAGATCCCAGAACCA | ACGAAGGCTCTGGTCCACTA |
| ENST00000417970 | ATCCGACCTTTTGTGACTGG | GCTTCCCAAAGTGCTGAGAC |
| NOTCH3 | CGTCAGTGTGAACTCCTCTCC | CCAGGTTGGTGCAGATACCAT |
| LOXL2 | ATGACAGCTGGCCCCATTAC | GGAGAAGTCGTCATCGCACA |
| PFK-1 | GGCATGGAAAGCCTATCT | CCATACCCATCTTGCTACTCA |
| RHOB | GACTACCTCGAGTGCTCTGC | TAGCACCTTGCAGCAGTTGA |
| KDR | CGGTCAACAAAGTCGGGAGA | CAGTGCACCACAAAGACACG |
| COL4A2 | CAACCCTGGTGATGTCTGCT | CGGCTGATGTAGGGCTTGAT |
| COL18A1 | CCACCATCTTCAGAGCGCA | ATCTGGCCCAAAGACGTAGG |
| AIMP1 | GGTCCTCCGCTTCATGATTTTC | GATTTGATCTGCCTCTGCACC |
| ACVRL1 | CTGCTGATGGCCTTGGTGA | AGAGCTCCCTGTGCAAGTTC |
| THY1 | CAGCATCGCTCTCCTGCTAA | ACTGGATGGGTGAACTGCTG |
| CUL5 | TGCGCCCGATTGTTTTGAAG | GCTGGGCCTTTATCATCCCA |
| MCAM | ACAGCACCTCCACAGAGAGA | TTCGCTCTTACGAGACGGG |
