# Supplementary material for: Microarray expression profile of mRNAs and long noncoding RNAs and the potential role of PFK-1 in infantile hemangioma
Source: Cell Div. 2021 Jan 11;16:1. doi: 10.1186/s13008-020-00069-y (PMC7802351; doi:10.1186/s13008-020-00069-y)
Supplement: Supplementary file 3 — Additional file 3: Table S3. Differentially expressed lncRNAs between proliferating and involuting infantile hemangioma. [file 13008_2020_69_MOESM3_ESM.docx]

**Table S3.** Differentially expressed lncRNAs between proliferating and involuting infantile hemangioma

| **LncRNA ID** | **Database Source** | **Fold Change** | **P-value** | **Feature** | **Rank** |
| --- | --- | --- | --- | --- | --- |
| TCONS_00000305-XLOC_000397 | Rinn lincRNA | -2.123991 | 3.50E-05 | Down | 1 |
| n334063 | NONCODE | 2.001631 | 4.80E-05 | Up | 2 |
| n333116 | NONCODE | 2.445146 | 5.60E-05 | Up | 3 |
| n341453 | NONCODE | -2.830943 | 8.00E-05 | Down | 4 |
| n333411 | NONCODE | -2.298218 | 8.30E-05 | Down | 5 |
| n410169 | NONCODE | -1.956713 | 0.000138 | Down | 6 |
| n342353 | NONCODE | -1.860167 | 0.000202 | Down | 7 |
| n335587 | NONCODE | 1.516683 | 0.00021 | Up | 8 |
| n335269 | NONCODE | -1.674877 | 0.000327 | Down | 9 |
| TCONS_l2_00001442-XLOC_l2_001047 | Broad TUCP | -1.511823 | 0.000417 | Down | 10 |
| n341432 | NONCODE | 2.612603 | 0.000468 | Up | 11 |
| n407167 | NONCODE | -1.523777 | 0.000469 | Down | 12 |
| NR_003674 | RefSeq | -1.579734 | 0.000486 | Down | 13 |
| n342217 | NONCODE | -1.677187 | 0.000507 | Down | 14 |
| ENST00000408086 | ENSEMBL | -1.553432 | 0.00056 | Down | 15 |
| n339835 | NONCODE | -1.505152 | 0.000579 | Down | 16 |
| n335088 | NONCODE | 1.602067 | 0.000595 | Up | 17 |
| n341422 | NONCODE | -2.779053 | 6.00E-04 | Down | 18 |
| TCONS_l2_00014894-XLOC_l2_008260 | Broad TUCP | -1.731534 | 0.000637 | Down | 19 |
| NR_003704 | RefSeq | 1.618506 | 0.000638 | Up | 20 |
| n339122 | NONCODE | -1.69899 | 0.000663 | Down | 21 |
| n342007 | NONCODE | 1.649037 | 0.000677 | Up | 22 |
| n341434 | NONCODE | 1.667257 | 0.000686 | Up | 23 |
| n337816 | NONCODE | -1.907751 | 7.00E-04 | Down | 24 |
| TCONS_00000324-XLOC_000441 | Rinn lincRNA | -1.533167 | 0.000736 | Down | 25 |
| n410638 | NONCODE | -1.874007 | 0.000784 | Down | 26 |
| n343017 | NONCODE | -1.916429 | 0.000812 | Down | 27 |
| n335702 | NONCODE | 1.537707 | 0.000827 | Up | 28 |
| TCONS_00002232-XLOC_000566 | Rinn lincRNA | -1.850101 | 0.000849 | Down | 29 |
| n342783 | NONCODE | -2.260328 | 0.000923 | Down | 30 |
| n334130 | NONCODE | -3.289001 | 0.000936 | Down | 31 |
| n406461 | NONCODE | -1.741215 | 0.00107 | Down | 32 |
| n409356 | NONCODE | -1.790054 | 0.001077 | Down | 33 |
| TCONS_l2_00030769-XLOC_l2_015655 | Broad TUCP | -1.584504 | 0.001155 | Down | 34 |
| n407424 | NONCODE | -1.572611 | 0.001157 | Down | 35 |
| n338024 | NONCODE | -1.802169 | 0.001184 | Down | 36 |
| NR_001588 | RefSeq | -1.777439 | 0.001197 | Down | 37 |
| NR_001293 | RefSeq | -1.775363 | 0.001259 | Down | 38 |
| n342711 | NONCODE | -1.527237 | 0.001267 | Down | 39 |
| TCONS_l2_00028762-XLOC_l2_014816 | Broad TUCP | -2.088036 | 0.001339 | Down | 40 |
| ENST00000437833 | ENSEMBL | -1.557896 | 0.00138 | Down | 41 |
| ENST00000340708 | ENSEMBL | -1.703328 | 0.001445 | Down | 42 |
| n333357 | NONCODE | -1.719807 | 0.00147 | Down | 43 |
| n408209 | NONCODE | -1.613397 | 0.001472 | Down | 44 |
| n407869 | NONCODE | -1.839413 | 0.001508 | Down | 45 |
| n335635 | NONCODE | -2.093126 | 0.001512 | Down | 46 |
| n342399 | NONCODE | 1.634329 | 0.001617 | Up | 47 |
| n408151 | NONCODE | -2.777391 | 0.001618 | Down | 48 |
| TCONS_l2_00030950-XLOC_l2_015963 | Broad TUCP | -1.895049 | 0.001759 | Down | 49 |
| NR_037879 | RefSeq | -1.629302 | 0.001829 | Down | 50 |
| NR_029410 | RefSeq | -1.660024 | 0.001841 | Down | 51 |
| n377717 | NONCODE | -1.778317 | 0.001909 | Down | 52 |
| n342877 | NONCODE | 1.545797 | 0.001973 | Up | 53 |
| ENST00000453508 | ENSEMBL | -1.65067 | 0.001995 | Down | 54 |
| n334786 | NONCODE | -1.82141 | 0.001997 | Down | 55 |
| TCONS_l2_00029300-XLOC_l2_015184 | Broad TUCP | -1.517729 | 0.001999 | Down | 56 |
| NR_002743 | RefSeq | -1.70872 | 0.002062 | Down | 57 |
| TCONS_00011942-XLOC_005452 | Rinn lincRNA | -1.656151 | 0.002076 | Down | 58 |
| n341598 | NONCODE | -3.500217 | 0.002157 | Down | 59 |
| n341264 | NONCODE | -2.867811 | 0.002184 | Down | 60 |
| TCONS_00020660-XLOC_009961 | Rinn lincRNA | 1.892177 | 0.002282 | Up | 61 |
| NR_030406 | RefSeq | 1.619264 | 0.002484 | Up | 62 |
| n342961 | NONCODE | 1.834266 | 0.002809 | Up | 63 |
| n333543 | NONCODE | 1.728863 | 0.002983 | Up | 64 |
| n335471 | NONCODE | 1.643517 | 0.003035 | Up | 65 |
| ENST00000411386 | ENSEMBL | 1.87732 | 0.003042 | Up | 66 |
| n338505 | NONCODE | 1.573171 | 0.003059 | Up | 67 |
| ENST00000408244 | ENSEMBL | 1.547368 | 0.003129 | Up | 68 |
| n337481 | NONCODE | 1.993223 | 0.003857 | Up | 69 |
| n340426 | NONCODE | 1.819108 | 0.004053 | Up | 70 |
| TCONS_00000027-XLOC_001272 | Rinn lincRNA | 1.977336 | 0.004553 | Up | 71 |
| n335092 | NONCODE | 1.511451 | 0.004636 | Up | 72 |
| ENST00000410589 | ENSEMBL | 1.63015 | 0.004939 | Up | 73 |
| n336986 | NONCODE | 1.644035 | 0.005051 | Up | 74 |
| ENST00000401378 | ENSEMBL | 1.509728 | 0.005687 | Up | 75 |
| n335645 | NONCODE | 1.940263 | 0.00581 | Up | 76 |
| NR_015407 | RefSeq | 1.88197 | 0.005856 | Up | 77 |
| ENST00000401125 | ENSEMBL | 1.739239 | 0.005994 | Up | 78 |
| n335708 | NONCODE | 1.771642 | 0.006317 | Up | 79 |
| n334214 | NONCODE | 2.309861 | 0.006359 | Up | 80 |
| n334398 | NONCODE | 3.244243 | 0.006409 | Up | 81 |
| n337901 | NONCODE | 1.749538 | 0.008656 | Up | 82 |
| n338280 | NONCODE | 1.515963 | 0.009182 | Up | 83 |
| TCONS_00029742-XLOC_014415 | Rinn lincRNA | 1.650116 | 0.009305 | Up | 84 |
| n342692 | NONCODE | 1.671106 | 0.009378 | Up | 85 |
| n410114 | NONCODE | 1.520099 | 0.009505 | Up | 86 |
| ENST00000401135 | ENSEMBL | 1.562554 | 0.00965 | Up | 87 |
| ENST00000458852 | ENSEMBL | 1.684903 | 0.009749 | Up | 88 |
| n341933 | NONCODE | 1.54845 | 0.009864 | Up | 89 |
| n332777 | NONCODE | 1.509588 | 0.010122 | Up | 90 |
| TCONS_00028418-XLOC_013771 | Rinn lincRNA | 1.778904 | 0.010611 | Up | 91 |
| n335203 | NONCODE | 1.816812 | 0.011099 | Up | 92 |
| n338957 | NONCODE | 1.762711 | 0.011337 | Up | 93 |
| ENST00000408817 | ENSEMBL | 1.562668 | 0.011501 | Up | 94 |
| n377787 | NONCODE | 1.636286 | 0.012087 | Up | 95 |
| n333545 | NONCODE | 1.582758 | 0.012845 | Up | 96 |
| ENST00000540136 | ENSEMBL | 1.608562 | 0.013265 | Up | 97 |
| ENST00000408353 | ENSEMBL | 2.052606 | 0.013349 | Up | 98 |
| n344651 | NONCODE | 1.587426 | 0.013494 | Up | 99 |
| TCONS_00012093-XLOC_005623 | Rinn lincRNA | 1.644268 | 0.013818 | Up | 100 |
| TCONS_l2_00011953-XLOC_l2_006397 | Broad TUCP | 1.512375 | 0.01386 | Up | 101 |
| n345837 | NONCODE | 1.530872 | 0.014632 | Up | 102 |
| ENST00000417970 | ENSEMBL | 1.515007 | 0.014714 | Up | 103 |
| n410469 | NONCODE | 1.546134 | 0.015003 | Up | 104 |
| n342556 | NONCODE | 1.653443 | 0.015088 | Up | 105 |
| ENST00000401296 | ENSEMBL | 1.703096 | 0.015091 | Up | 106 |
| NR_015395 | RefSeq | 1.529854 | 0.016239 | Up | 107 |
| ENST00000408883 | ENSEMBL | 1.850772 | 0.016252 | Up | 108 |
| ENST00000525418 | ENSEMBL | 1.629272 | 0.016655 | Up | 109 |
| ENST00000408692 | ENSEMBL | 2.0277 | 0.016739 | Up | 110 |
| NR_024204 | RefSeq | 1.571805 | 0.017023 | Up | 111 |
| NR_029704 | RefSeq | 1.564024 | 0.017119 | Up | 112 |
| ENST00000408306 | ENSEMBL | 1.839422 | 0.018015 | Up | 113 |
| TCONS_l2_00022340-XLOC_l2_011607 | Broad TUCP | 1.727583 | 0.018029 | Up | 114 |
| n333334 | NONCODE | 1.892021 | 0.018135 | Up | 115 |
| ENST00000428911 | ENSEMBL | 1.581671 | 0.018167 | Up | 116 |
| ENST00000401297 | ENSEMBL | 1.540464 | 0.019053 | Up | 117 |
| n408884 | NONCODE | 1.750886 | 0.019682 | Up | 118 |
| n336980 | NONCODE | 1.649028 | 0.020298 | Up | 119 |
| ENST00000401362 | ENSEMBL | 1.939533 | 0.021198 | Up | 120 |
| ENST00000401257 | ENSEMBL | 1.842352 | 0.022755 | Up | 121 |
| n334841 | NONCODE | 1.674029 | 0.022975 | Up | 122 |
| ENST00000401371 | ENSEMBL | 1.564659 | 0.023399 | Up | 123 |
| n335785 | NONCODE | 2.088947 | 0.02344 | Up | 124 |
| n335743 | NONCODE | 1.745571 | 0.024213 | Up | 125 |
| n341258 | NONCODE | 1.680217 | 0.024559 | Up | 126 |
| TCONS_00024775-XLOC_012050 | Rinn lincRNA | 1.584977 | 0.025866 | Up | 127 |
| ENST00000401274 | ENSEMBL | 1.646487 | 0.025889 | Up | 128 |
| n338288 | NONCODE | 1.679238 | 0.026167 | Up | 129 |
| ENST00000401164 | ENSEMBL | 1.924029 | 0.02638 | Up | 130 |
| n385114 | NONCODE | 1.901713 | 0.026762 | Up | 131 |
| n335566 | NONCODE | 1.605935 | 0.028473 | Up | 132 |
| ENST00000401185 | ENSEMBL | 1.564885 | 0.028915 | Up | 133 |
| TCONS_00017433-XLOC_008015 | Rinn lincRNA | 1.824321 | 0.03174 | Up | 134 |
| n332696 | NONCODE | 1.906359 | 0.03213 | Up | 135 |
| ENST00000408685 | ENSEMBL | 1.593513 | 0.0332 | Up | 136 |
| ENST00000408617 | ENSEMBL | 1.86486 | 0.033881 | Up | 137 |
| ENST00000401269 | ENSEMBL | 1.524637 | 0.034083 | Up | 138 |
| ENST00000401115 | ENSEMBL | 1.768469 | 0.037156 | Up | 139 |
| n387037 | NONCODE | 1.857163 | 0.039104 | Up | 140 |
| ENST00000408308 | ENSEMBL | 1.629356 | 0.040691 | Up | 141 |
| NR_030619 | RefSeq | 1.756813 | 0.040762 | Up | 142 |
| n340496 | NONCODE | 1.636351 | 0.041114 | Up | 143 |
| n409332 | NONCODE | 1.502411 | 0.043325 | Up | 144 |
| n384299 | NONCODE | 1.622928 | 0.043442 | Up | 145 |
| n345882 | NONCODE | 1.600473 | 0.043586 | Up | 146 |
