# Supplementary material for: Microarray expression profile of mRNAs and long noncoding RNAs and the potential role of PFK-1 in infantile hemangioma
Source: Cell Div. 2021 Jan 11;16:1. doi: 10.1186/s13008-020-00069-y (PMC7802351; doi:10.1186/s13008-020-00069-y)
Supplement: Supplementary file 4 — Additional file 4: Table S4. Differentially expressed mRNAs between proliferating and involuting infantile hemangioma. [file 13008_2020_69_MOESM4_ESM.docx]

**Table S4.** Differentially expressed mRNAs between proliferating and involuting infantile hemangioma

| **Gene Symbol** | **Fold Change** | **P-value** | **Gene feature** | **Rank** |
| --- | --- | --- | --- | --- |
| NOTCH3 | 2.211008 | 0.000108 | Up | 1 |
| LYPLAL1 | -2.003661 | 0.00012 | Down | 2 |
| PFK1 | 1.565332 | 0.000133 | Up | 3 |
| LOXL2 | 1.852747 | 0.000137 | Up | 4 |
| SNCA | -1.617441 | 0.00014 | Down | 5 |
| DOCK6 | 1.544228 | 0.000172 | Up | 6 |
| UGP2 | -1.824338 | 0.000175 | Down | 7 |
| HSDL2 | -1.98112 | 0.000181 | Down | 8 |
| COL18A1 | 1.582594 | 0.000192 | Up | 9 |
| PDGFRB | 1.982871 | 0.000202 | Up | 10 |
| TATDN3 | -1.530694 | 0.000259 | Down | 11 |
| PXDN | 1.636952 | 0.000297 | Up | 12 |
| CCDC91 | -1.592118 | 0.000348 | Down | 13 |
| OSGIN2 | -2.072383 | 0.000366 | Down | 14 |
| NUDT12 | -1.884237 | 0.000398 | Down | 15 |
| HINT3 | -2.359098 | 4.00E-04 | Down | 16 |
| ADAMTS4 | 1.852712 | 0.000401 | Up | 17 |
| ZDHHC2 | -2.49763 | 0.000429 | Down | 18 |
| SCP2 | -1.650027 | 0.000431 | Down | 19 |
| HSD11B2 | 1.541955 | 0.00046 | Up | 20 |
| MAMDC2 | -1.861169 | 0.00049 | Down | 21 |
| GATM | -1.745559 | 0.000516 | Down | 22 |
| MNAT1 | -1.927536 | 0.000517 | Down | 23 |
| PCMTD1 | -1.790591 | 0.00052 | Down | 24 |
| KLHDC1 | -1.673151 | 0.000524 | Down | 25 |
| TDRD3 | -1.505693 | 0.000554 | Down | 26 |
| CD248 | 1.648343 | 0.000584 | Up | 27 |
| ACAN | 1.704538 | 6.00E-04 | Up | 28 |
| FLNA | 1.699094 | 0.000618 | Up | 29 |
| N4BP2L2 | -1.839181 | 0.000654 | Down | 30 |
| WBP4 | -1.966638 | 0.000655 | Down | 31 |
| MYO1C | 1.594664 | 0.000668 | Up | 32 |
| CPED1 | -1.532991 | 0.000673 | Down | 33 |
| SPRY4 | 1.802916 | 0.000716 | Up | 34 |
| LYRM5 | -1.819563 | 0.000727 | Down | 35 |
| COL4A2 | 2.395693 | 0.000766 | Up | 36 |
| RERGL | -1.925621 | 0.000768 | Down | 37 |
| VASP | 1.55059 | 0.00077 | Up | 38 |
| DNAJC19 | -1.694298 | 0.000791 | Down | 39 |
| ENG | 1.567089 | 0.00081 | Up | 40 |
| NOTCH1 | 1.505646 | 0.000815 | Up | 41 |
| USP53 | -2.044246 | 0.000847 | Down | 42 |
| IL18 | -2.070839 | 0.000861 | Down | 43 |
| NID1 | 1.924221 | 0.000941 | Up | 44 |
| FXR1 | -1.902593 | 0.000973 | Down | 45 |
| TLN1 | 1.548241 | 0.001011 | Up | 46 |
| PLXND1 | 1.816676 | 0.001016 | Up | 47 |
| SUCLA2 | -1.703151 | 0.001022 | Down | 48 |
| TIMM21 | -1.574343 | 0.001031 | Down | 49 |
| PLA2G12A | -1.672222 | 0.001095 | Down | 50 |
| SESN1 | -1.876584 | 0.001119 | Down | 51 |
| MMP14 | 1.646649 | 0.001126 | Up | 52 |
| TAP1 | 1.50674 | 0.00115 | Up | 53 |
| UBE2B | -1.61738 | 0.001153 | Down | 54 |
| OLFML2A | 1.968613 | 0.001166 | Up | 55 |
| NIPSNAP3B | -2.239202 | 0.001172 | Down | 56 |
| RPS6KA5 | -1.593255 | 0.001187 | Down | 57 |
| PLEKHG2 | 1.557095 | 0.001214 | Up | 58 |
| COLGALT1 | 1.541408 | 0.001216 | Up | 59 |
| KIRREL | 1.722936 | 0.001234 | Up | 60 |
| PVRL2 | 1.624807 | 0.001257 | Up | 61 |
| ACOT13 | -1.586711 | 0.001283 | Down | 62 |
| PTGR2 | -1.717978 | 0.001284 | Down | 63 |
| FMO2 | -2.699681 | 0.001388 | Down | 64 |
| UQCRB | -1.620405 | 0.001389 | Down | 65 |
| DLL4 | 1.562826 | 0.001405 | Up | 66 |
| DLD | -1.877071 | 0.001431 | Down | 67 |
| GIN1 | -1.797072 | 0.001459 | Down | 68 |
| OLFM2 | 1.620033 | 0.001473 | Up | 69 |
| CDC37L1 | -1.80723 | 0.001486 | Down | 70 |
| LRRK2 | -1.80695 | 0.001503 | Down | 71 |
| FKBP3 | -1.942647 | 0.001508 | Down | 72 |
| PTBP1 | 1.555737 | 0.001539 | Up | 73 |
| LDLR | 1.622539 | 0.001555 | Up | 74 |
| MPC1 | -1.550295 | 0.001562 | Down | 75 |
| NDUFA4L2 | 1.500358 | 0.001565 | Up | 76 |
| LYRM7 | -1.711836 | 0.001571 | Down | 77 |
| EDA2R | -1.93064 | 0.001582 | Down | 78 |
| GHITM | -1.635521 | 0.001645 | Down | 79 |
| TATDN1 | -1.502379 | 0.001684 | Down | 80 |
| ACAT1 | -2.001003 | 0.001714 | Down | 81 |
| GPR4 | 1.642676 | 0.00175 | Up | 82 |
| ZC3H6 | -1.620529 | 0.001774 | Down | 83 |
| DBT | -1.624307 | 0.001784 | Down | 84 |
| ZNF680 | -1.623717 | 0.001789 | Down | 85 |
| GOLGA4 | -1.65164 | 0.00179 | Down | 86 |
| ALDH1A1 | -2.137581 | 0.001815 | Down | 87 |
| TINAGL1 | 1.748604 | 0.001823 | Up | 88 |
| ARHGEF17 | 1.805288 | 0.001962 | Up | 89 |
| ADAMTS12 | 1.622087 | 0.002003 | Up | 90 |
| ROBO4 | 1.677708 | 0.002004 | Up | 91 |
| ITGA5 | 1.859768 | 0.002024 | Up | 92 |
| OAS3 | 1.503998 | 0.002071 | Up | 93 |
| RAB4A | -1.66165 | 0.002129 | Down | 94 |
| KLHL24 | -1.791561 | 0.002165 | Down | 95 |
| CFL2 | -2.820907 | 0.002205 | Down | 96 |
| GBE1 | -1.619331 | 0.002213 | Down | 97 |
| DTWD2 | -1.59851 | 0.002218 | Down | 98 |
| SGCB | -1.56745 | 0.002296 | Down | 99 |
| MTIF2 | -1.665926 | 0.002366 | Down | 100 |
| COL4A1 | 2.501585 | 0.002376 | Up | 101 |
| MOCS2 | -1.511773 | 0.002407 | Down | 102 |
| HIST1H2BB | 1.605249 | 0.002423 | Up | 103 |
| DKK2 | -1.502712 | 0.002554 | Down | 104 |
| GTPBP8 | -1.733933 | 0.002603 | Down | 105 |
| NDUFB6 | -1.701157 | 0.002604 | Down | 106 |
| ADCYAP1R1 | 1.64681 | 0.002608 | Up | 107 |
| COX7C | -1.549407 | 0.002636 | Down | 108 |
| COL6A2 | 1.539987 | 0.002659 | Up | 109 |
| ESAM | 1.583093 | 0.00267 | Up | 110 |
| MYH9 | 1.671994 | 0.002674 | Up | 111 |
| PYROXD1 | -1.832931 | 0.002701 | Down | 112 |
| MSTN | -2.624716 | 0.00271 | Down | 113 |
| CADM2 | -1.924605 | 0.002714 | Down | 114 |
| SEMA5A | 1.806387 | 0.002725 | Up | 115 |
| WWP1 | -1.798275 | 0.002806 | Down | 116 |
| PM20D2 | -1.96314 | 0.002824 | Down | 117 |
| CLK4 | -1.75397 | 0.002843 | Down | 118 |
| UBE2D1 | -2.344914 | 0.002874 | Down | 119 |
| PRDX3 | -1.702882 | 0.002932 | Down | 120 |
| EBAG9 | -1.661359 | 0.002941 | Down | 121 |
| MCAM | 1.991666 | 0.002979 | Up | 122 |
| MRPS36 | -1.505909 | 0.003004 | Down | 123 |
| CCNG1 | -1.771938 | 0.003071 | Down | 124 |
| MPP6 | -1.509348 | 0.003076 | Down | 125 |
| RRM2B | -1.920731 | 0.003121 | Down | 126 |
| ACADSB | -1.88916 | 0.003122 | Down | 127 |
| DDIT4L | -1.655266 | 0.003168 | Down | 128 |
| CCL3L3 | 1.632855 | 0.003171 | Up | 129 |
| SOX4 | 1.537453 | 0.00325 | Up | 130 |
| LAMB1 | 1.576653 | 0.003281 | Up | 131 |
| HSF2 | -2.059508 | 0.003294 | Down | 132 |
| SBDS | -1.584871 | 0.003323 | Down | 133 |
| ATG14 | -1.763215 | 0.003463 | Down | 134 |
| COPS2 | -1.874503 | 0.003477 | Down | 135 |
| SERPINH1 | 1.530073 | 0.003503 | Up | 136 |
| AKAP9 | -1.547432 | 0.00352 | Down | 137 |
| COQ5 | -1.513142 | 0.003596 | Down | 138 |
| ISCU | -1.515214 | 0.003717 | Down | 139 |
| NDUFS4 | -1.572339 | 0.003776 | Down | 140 |
| ZNF25 | -1.696015 | 0.003859 | Down | 141 |
| ANPEP | 1.510058 | 0.003903 | Up | 142 |
| NT5C3A | -1.688366 | 0.003914 | Down | 143 |
| ACADM | -1.721258 | 0.003933 | Down | 144 |
| NEXN | -3.018095 | 0.003949 | Down | 145 |
| DZIP3 | -1.725692 | 0.003983 | Down | 146 |
| ZFP36 | 1.647599 | 0.003999 | Up | 147 |
| ARL5A | -1.559184 | 0.004008 | Down | 148 |
| KIF21A | -1.518583 | 0.004033 | Down | 149 |
| USP25 | -1.680214 | 0.004051 | Down | 150 |
| BARD1 | -1.571184 | 0.004053 | Down | 151 |
| ATP6V1D | -1.620077 | 0.004137 | Down | 152 |
| PPP3CB | -1.672396 | 0.004144 | Down | 153 |
| HSPA4L | -1.571481 | 0.004189 | Down | 154 |
| ZNF181 | -1.573305 | 0.004224 | Down | 155 |
| CNKSR2 | -1.770252 | 0.004225 | Down | 156 |
| TRMT1L | -1.615601 | 0.00435 | Down | 157 |
| DNAJC21 | -1.622402 | 0.004351 | Down | 158 |
| LACTB | -1.553993 | 0.004376 | Down | 159 |
| CAB39 | -1.644373 | 0.004608 | Down | 160 |
| AKTIP | -1.700918 | 0.004618 | Down | 161 |
| GPER | 1.500873 | 0.004621 | Up | 162 |
| NDUFB5 | -1.639639 | 0.004704 | Down | 163 |
| TGFB1I1 | 1.760263 | 0.004728 | Up | 164 |
| EIF1AY | -2.730232 | 0.004758 | Down | 165 |
| CISD1 | -1.944267 | 0.004835 | Down | 166 |
| SLC25A46 | -1.534094 | 0.004876 | Down | 167 |
| HBS1L | -1.66516 | 0.004877 | Down | 168 |
| TM6SF1 | -1.687833 | 0.00496 | Down | 169 |
| GCFC2 | -1.5764 | 0.005009 | Down | 170 |
| PPP1CB | -1.590005 | 0.005132 | Down | 171 |
| NID2 | 1.722497 | 0.005138 | Up | 172 |
| RABGGTB | -1.569533 | 0.005147 | Down | 173 |
| ABCA10 | -1.850534 | 0.005202 | Down | 174 |
| HMGN3 | -1.523173 | 0.005273 | Down | 175 |
| GNPAT | -1.726302 | 0.005346 | Down | 176 |
| ITGBL1 | -1.708382 | 0.005474 | Down | 177 |
| PPP1CC | -1.611769 | 0.005672 | Down | 178 |
| TSPAN15 | 1.669188 | 0.00574 | Up | 179 |
| AASS | -1.518535 | 0.005751 | Down | 180 |
| USO1 | -1.600265 | 0.005811 | Down | 181 |
| VPS13A | -1.780173 | 0.005872 | Down | 182 |
| CAMK2D | -1.532938 | 0.005911 | Down | 183 |
| SAR1B | -1.6394 | 0.005918 | Down | 184 |
| USP15 | -1.777344 | 0.005939 | Down | 185 |
| CTNNA3 | -1.924035 | 0.006013 | Down | 186 |
| BTBD1 | -2.022647 | 0.006037 | Down | 187 |
| ASNSD1 | -1.70752 | 0.006111 | Down | 188 |
| NADK2 | -1.523914 | 0.006116 | Down | 189 |
| BNIP3 | -1.556977 | 0.006149 | Down | 190 |
| RHOB | 1.811876 | 0.006159 | Up | 191 |
| ME1 | -2.388245 | 0.006215 | Down | 192 |
| HADHB | -1.714643 | 0.006305 | Down | 193 |
| CLIC2 | -1.997263 | 0.006373 | Down | 194 |
| STRADB | -1.901663 | 0.006499 | Down | 195 |
| GBAS | -1.953689 | 0.006522 | Down | 196 |
| ADAM12 | 1.524716 | 0.006767 | Up | 197 |
| TCP11L2 | -1.715663 | 0.006846 | Down | 198 |
| DNAJB4 | -1.655081 | 0.006873 | Down | 199 |
| ADAL | -1.962363 | 0.00693 | Down | 200 |
| VAMP4 | -1.734838 | 0.00699 | Down | 201 |
| EMB | -1.692381 | 0.007165 | Down | 202 |
| ZBTB44 | -1.614757 | 0.0072 | Down | 203 |
| TOB1 | -1.805708 | 0.007219 | Down | 204 |
| CUL5 | -1.900919 | 0.007236 | Down | 205 |
| DMD | -2.289538 | 0.007258 | Down | 206 |
| KPNA5 | -1.765594 | 0.007266 | Down | 207 |
| S1PR3 | 1.946989 | 0.007405 | Up | 208 |
| CREG1 | -1.522206 | 0.007453 | Down | 209 |
| CYCS | -1.645191 | 0.007507 | Down | 210 |
| RAVER2 | -1.612135 | 0.007642 | Down | 211 |
| MMADHC | -1.625486 | 0.007678 | Down | 212 |
| THY1 | 1.920826 | 0.007751 | Up | 213 |
| TMEM65 | -1.602733 | 0.007754 | Down | 214 |
| FOSB | 1.675378 | 0.007814 | Up | 215 |
| MTM1 | -1.532424 | 0.007819 | Down | 216 |
| TMEM38B | -2.344927 | 0.008044 | Down | 217 |
| MYOZ2 | -5.902445 | 0.008275 | Down | 218 |
| LIPG | 1.577442 | 0.008359 | Up | 219 |
| CAPZA2 | -1.727896 | 0.008411 | Down | 220 |
| TAPT1 | -1.543905 | 0.008416 | Down | 221 |
| PPP1R3C | -2.708913 | 0.008451 | Down | 222 |
| COX20 | -1.614284 | 0.008488 | Down | 223 |
| GUF1 | -1.753363 | 0.008556 | Down | 224 |
| PCM1 | -1.589897 | 0.008589 | Down | 225 |
| AGL | -2.833037 | 0.008609 | Down | 226 |
| PHTF2 | -1.904935 | 0.00861 | Down | 227 |
| PRKAA2 | -5.020766 | 0.008712 | Down | 228 |
| SLC2A12 | -1.705585 | 0.008733 | Down | 229 |
| BAG2 | -1.664933 | 0.008755 | Down | 230 |
| MYOM1 | -2.666035 | 0.008795 | Down | 231 |
| ANGPTL1 | -1.895571 | 0.008806 | Down | 232 |
| CRBN | -1.544529 | 0.008857 | Down | 233 |
| UHRF1BP1L | -1.547484 | 0.008923 | Down | 234 |
| SLMAP | -1.799965 | 0.008952 | Down | 235 |
| TARSL2 | -1.728785 | 0.00896 | Down | 236 |
| UBE3A | -1.574994 | 0.008981 | Down | 237 |
| BLOC1S2 | -1.561574 | 0.009376 | Down | 238 |
| ACTN4 | 1.586096 | 0.009545 | Up | 239 |
| PLVAP | 1.919827 | 0.00958 | Up | 240 |
| PAIP2B | -2.924361 | 0.009618 | Down | 241 |
| TNIK | -1.777253 | 0.009629 | Down | 242 |
| LMBRD1 | -1.595359 | 0.009802 | Down | 243 |
| PSMD12 | -1.714035 | 0.009827 | Down | 244 |
| P4HA1 | -1.562494 | 0.009845 | Down | 245 |
| ZNF106 | -2.129215 | 0.009862 | Down | 246 |
| HTATSF1 | -1.637718 | 0.009946 | Down | 247 |
| KTN1 | -1.68247 | 0.010063 | Down | 248 |
| TTC33 | -1.896312 | 0.010147 | Down | 249 |
| MIOS | -1.508716 | 0.010161 | Down | 250 |
| MYO10 | 1.535942 | 0.010193 | Up | 251 |
| VASN | 1.601435 | 0.01035 | Up | 252 |
| AS3MT | -1.514148 | 0.010369 | Down | 253 |
| C18orf25 | -1.583077 | 0.010372 | Down | 254 |
| MRS2 | -1.557946 | 0.010417 | Down | 255 |
| CCL3 | 1.687333 | 0.010621 | Up | 256 |
| POLB | -1.55754 | 0.010639 | Down | 257 |
| NDUFAF1 | -1.517614 | 0.010843 | Down | 258 |
| KAT2B | -1.614584 | 0.01104 | Down | 259 |
| ABCB7 | -1.508848 | 0.011088 | Down | 260 |
| CACNA2D1 | -2.252442 | 0.011104 | Down | 261 |
| PKP2 | -1.658897 | 0.011131 | Down | 262 |
| SNX16 | -1.737484 | 0.01115 | Down | 263 |
| RAB12 | -1.561086 | 0.011203 | Down | 264 |
| RRAS2 | -1.506683 | 0.011215 | Down | 265 |
| ABHD10 | -1.670946 | 0.011383 | Down | 266 |
| UBR3 | -1.804681 | 0.011422 | Down | 267 |
| ARHGAP1 | 1.503865 | 0.011458 | Up | 268 |
| TMEM182 | -2.948632 | 0.011545 | Down | 269 |
| CRYZ | -1.646267 | 0.011588 | Down | 270 |
| DHRS7 | -1.529333 | 0.011594 | Down | 271 |
| HIST1H2BM | 1.558631 | 0.011762 | Up | 272 |
| TRIM23 | -1.551266 | 0.011797 | Down | 273 |
| MXRA5 | 1.715517 | 0.0119 | Up | 274 |
| AIMP1 | -1.658677 | 0.011927 | Down | 275 |
| SCN7A | -2.69102 | 0.011999 | Down | 276 |
| RWDD2B | -1.503017 | 0.012029 | Down | 277 |
| MRPS30 | -1.518261 | 0.012101 | Down | 278 |
| CASQ2 | -2.444453 | 0.012142 | Down | 279 |
| TCAIM | -1.71114 | 0.012143 | Down | 280 |
| CCDC43 | -1.625237 | 0.012199 | Down | 281 |
| CCDC141 | -1.543191 | 0.012321 | Down | 282 |
| DDO | -1.591295 | 0.012504 | Down | 283 |
| BGN | 1.619226 | 0.012656 | Up | 284 |
| PDGFB | 1.544599 | 0.012704 | Up | 285 |
| DCUN1D1 | -1.673072 | 0.01271 | Down | 286 |
| ACTN1 | 1.506622 | 0.012742 | Up | 287 |
| ACTR6 | -1.787 | 0.012879 | Down | 288 |
| FAT1 | 1.877977 | 0.012943 | Up | 289 |
| TNN | 2.126704 | 0.013143 | Up | 290 |
| FSD1L | -1.756549 | 0.013146 | Down | 291 |
| EEA1 | -1.510489 | 0.013191 | Down | 292 |
| DIO2 | -1.660122 | 0.01326 | Down | 293 |
| IBTK | -1.602408 | 0.01327 | Down | 294 |
| EYA4 | -2.202467 | 0.013366 | Down | 295 |
| CPEB4 | -1.736599 | 0.013421 | Down | 296 |
| ZNF91 | -1.53373 | 0.013446 | Down | 297 |
| COPS4 | -1.521676 | 0.013599 | Down | 298 |
| LMBRD2 | -1.644826 | 0.013622 | Down | 299 |
| CHURC1 | -1.526012 | 0.013657 | Down | 300 |
| PPP1R2 | -1.554038 | 0.013675 | Down | 301 |
| EPDR1 | -1.848101 | 0.01377 | Down | 302 |
| VDAC3 | -1.525647 | 0.013818 | Down | 303 |
| PIAS2 | -1.522662 | 0.013857 | Down | 304 |
| CUL2 | -1.5414 | 0.013956 | Down | 305 |
| RBL1 | -1.542558 | 0.014013 | Down | 306 |
| FOPNL | -1.809162 | 0.014091 | Down | 307 |
| MLF1 | -2.652872 | 0.014123 | Down | 308 |
| NPR1 | 1.552684 | 0.01426 | Up | 309 |
| MOSPD1 | -1.606696 | 0.014336 | Down | 310 |
| PTP4A1 | -1.720613 | 0.014468 | Down | 311 |
| PLN | -2.220745 | 0.01453 | Down | 312 |
| OPA1 | -1.540141 | 0.014941 | Down | 313 |
| SCN4B | 1.546465 | 0.014974 | Up | 314 |
| KPNA4 | -1.808364 | 0.01513 | Down | 315 |
| AGTPBP1 | -1.525615 | 0.015206 | Down | 316 |
| ABCA5 | -1.780284 | 0.015303 | Down | 317 |
| MSI2 | -1.549554 | 0.015594 | Down | 318 |
| RBM18 | -1.538519 | 0.01614 | Down | 319 |
| STRN3 | -1.56145 | 0.016264 | Down | 320 |
| MET | -1.539784 | 0.016366 | Down | 321 |
| NRD1 | -1.521864 | 0.016434 | Down | 322 |
| POPDC3 | -2.655182 | 0.016491 | Down | 323 |
| CLIP1 | -1.728327 | 0.016499 | Down | 324 |
| NDUFAF4 | -1.650885 | 0.016568 | Down | 325 |
| ME2 | -1.609037 | 0.016769 | Down | 326 |
| NARG2 | -1.526019 | 0.016951 | Down | 327 |
| IGJ | -1.573789 | 0.01704 | Down | 328 |
| RPF2 | -1.563784 | 0.017069 | Down | 329 |
| IFI6 | 1.992818 | 0.017341 | Up | 330 |
| SEMA3C | -1.775287 | 0.017388 | Down | 331 |
| GKAP1 | -1.532123 | 0.017503 | Down | 332 |
| DEPTOR | -1.645408 | 0.017559 | Down | 333 |
| DCAF6 | -1.840911 | 0.01783 | Down | 334 |
| FBXO32 | -2.391975 | 0.017843 | Down | 335 |
| ZNF615 | -1.625645 | 0.017881 | Down | 336 |
| ASF1A | -1.521228 | 0.018016 | Down | 337 |
| ATP1B1 | -1.966945 | 0.01817 | Down | 338 |
| TFRC | -1.759252 | 0.018336 | Down | 339 |
| EGR1 | 1.775311 | 0.019313 | Up | 340 |
| CETP | 1.795975 | 0.01938 | Up | 341 |
| ANO1 | 1.644849 | 0.020025 | Up | 342 |
| MAGED2 | 1.592846 | 0.020726 | Up | 343 |
| AOC3 | 1.740179 | 0.021739 | Up | 344 |
| TBX2 | 1.52247 | 0.021752 | Up | 345 |
| MYL9 | 1.622037 | 0.02276 | Up | 346 |
| KDR | 2.478637 | 0.022919 | Up | 347 |
| HIST1H3B | 1.781797 | 0.023479 | Up | 348 |
| APLN | 2.69105 | 0.024146 | Up | 349 |
| ID3 | 1.513899 | 0.02432 | Up | 350 |
| HIST1H3F | 1.640511 | 0.024799 | Up | 351 |
| GJA5 | 1.62996 | 0.026459 | Up | 352 |
| ACVRL1 | 1.557758 | 0.026785 | Up | 353 |
| ACTG2 | 1.564071 | 0.026988 | Up | 354 |
| CXorf36 | 1.929454 | 0.027672 | Up | 355 |
| CD4 | 1.62901 | 0.027902 | Up | 356 |
| ELN | 1.559719 | 0.029079 | Up | 357 |
| CRIM1 | 1.56691 | 0.029153 | Up | 358 |
| ACTA2 | 1.609589 | 0.029662 | Up | 359 |
| ACLY | 1.698288 | 0.029672 | Up | 360 |
| SLIT3 | 1.516863 | 0.029994 | Up | 361 |
| FOLH1B | 1.665317 | 0.030038 | Up | 362 |
| HIST1H3J | 1.59328 | 0.030966 | Up | 363 |
| FAM101B | 1.827589 | 0.034734 | Up | 364 |
| PDE1B | 1.675166 | 0.035735 | Up | 365 |
| SLC38A11 | 2.280045 | 0.036597 | Up | 366 |
| MSN | 1.523705 | 0.036859 | Up | 367 |
| SLCO2A1 | 1.509705 | 0.037016 | Up | 368 |
| CYGB | 1.667798 | 0.03837 | Up | 369 |
| TAGLN | 1.543902 | 0.042189 | Up | 370 |
| COL1A1 | 1.530545 | 0.047257 | Up | 371 |
| FOLH1 | 1.60412 | 0.047541 | Up | 372 |
| EPAS1 | 1.803839 | 0.048336 | Up | 373 |
| CHRM4 | 1.507497 | 0.049587 | Up | 374 |
