# Supplementary material for: Microarray expression profile of mRNAs and long noncoding RNAs and the potential role of PFK-1 in infantile hemangioma
Source: Cell Div. 2021 Jan 11;16:1. doi: 10.1186/s13008-020-00069-y (PMC7802351; doi:10.1186/s13008-020-00069-y)
Supplement: Supplementary file 5 — Additional file 5: Table S5. GO analysis of differentially expressed mRNAs. [file 13008_2020_69_MOESM5_ESM.docx]

**Table S5.** GO analysis of differentially expressed mRNAs

| **GO ID** | **Go name** | **P-value** | **FDR** | **Rank** |
| --- | --- | --- | --- | --- |
| GO:0001525 | Angiogenesis | 3.73E-13 | 3.42E-10 | 1 |
| GO:0044281 | Small molecule metabolic process­­ | 4.51E-13 | 3.42E-10 | 2 |
| GO:0007155 | Cell adhesion | 1.73E-10 | 8.73E-08 | 3 |
| GO:0030335 | Positive regulation of cell migration | 6.26E-10 | 2.37E-07 | 4 |
| GO:0007411 | Axon guidance | 5.45E-09 | 1.65E-06 | 5 |
| GO:0007179 | Transforming growth factor beta receptor signaling pathway | 1.97E-07 | 4.65E-05 | 6 |
| GO:0001666 | Response to hypoxia | 2.16E-07 | 4.65E-05 | 7 |
| GO:0010881 | Regulation of cardiac muscle contraction by regulation of the release of sequestered calcium ion | 2.46E-07 | 4.65E-05 | 8 |
| GO:0030198 | Extracellular matrix organization | 6.08E-07 | 0.000102 | 9 |
| GO:0005978 | Glycogen biosynthetic process | 6.86E-07 | 0.000104 | 10 |
| GO:0008150 | Biological_process | 9.68E-07 | 0.000133 | 11 |
| GO:0001974 | Blood vessel remodeling | 1.16E-06 | 0.000146 | 12 |
| GO:0007050 | Cell cycle arrest | 3.38E-06 | 0.000394 | 13 |
| GO:0019048 | Virus-host interaction | 6.17E-06 | 0.000668 | 14 |
| GO:0045022 | Early endosome to late endosome transport | 7.00E-06 | 0.000695 | 15 |
| GO:0008333 | Endosome to lysosome transport | 7.33E-06 | 0.000695 | 16 |
| GO:0022617 | Extracellular matrix disassembly | 1.06E-05 | 0.000945 | 17 |
| GO:0006508 | Proteolysis | 1.15E-05 | 0.000969 | 18 |
| GO:0016337 | Cell-cell adhesion | 1.87E-05 | 0.001489 | 19 |
| GO:0045944 | Positive regulation of transcription from RNA polymerase II promoter | 2.72E-05 | 0.002057 | 20 |
| GO:0009083 | Branched-chain amino acid catabolic process | 2.89E-05 | 0.002057 | 21 |
| GO:0008285 | Negative regulation of cell proliferation | 2.98E-05 | 0.002057 | 22 |
| GO:0006120 | Mitochondrial electron transport, NADH to ubiquinone | 3.49E-05 | 0.00227 | 23 |
| GO:0006936 | Muscle contraction | 3.59E-05 | 0.00227 | 24 |
| GO:0030199 | Collagen fibril organization | 3.98E-05 | 0.002355 | 25 |
| GO:0006511 | Ubiquitin-dependent protein catabolic process | 4.11E-05 | 0.002355 | 26 |
| GO:0040012 | Regulation of locomotion | 4.19E-05 | 0.002355 | 27 |
| GO:0006979 | Response to oxidative stress | 5.35E-05 | 0.002899 | 28 |
| GO:0051764 | Actin crosslink formation | 6.67E-05 | 0.003487 | 29 |
| GO:0030336 | Negative regulation of cell migration | 7.17E-05 | 0.003509 | 30 |
| GO:0030574 | Collagen catabolic process | 7.17E-05 | 0.003509 | 31 |
| GO:0007264 | Small gtpase mediated signal transduction | 7.44E-05 | 0.003523 | 32 |
| GO:0050900 | Leukocyte migration | 7.77E-05 | 0.00357 | 33 |
| GO:0044255 | Cellular lipid metabolic process | 8.65E-05 | 0.003858 | 34 |
| GO:0010880 | Regulation of release of sequestered calcium ion into cytosol by sarcoplasmic reticulum | 9.94E-05 | 0.004186 | 35 |
| GO:0015914 | Phospholipid transport | 9.94E-05 | 0.004186 | 36 |
| GO:0005977 | Glycogen metabolic process | 0.0001141 | 0.004677 | 37 |
| GO:0030217 | T cell differentiation | 0.000134 | 0.005346 | 38 |
| GO:0060314 | Regulation of ryanodine-sensitive calcium-release channel activity | 0.0001411 | 0.005361 | 39 |
| GO:0071506 | Cellular response to mycophenolic acid | 0.000145 | 0.005361 | 40 |
| GO:0072262 | Metanephric glomerular mesangial cell proliferation involved in metanephros development | 0.000145 | 0.005361 | 41 |
| GO:0048754 | Branching morphogenesis of an epithelial tube | 0.0001562 | 0.00564 | 42 |
| GO:0043066 | Negative regulation of apoptotic process | 0.0001687 | 0.005947 | 43 |
| GO:0016567 | Protein ubiquitination | 0.0001733 | 0.00597 | 44 |
| GO:0022904 | Respiratory electron transport chain | 0.0001953 | 0.006581 | 45 |
| GO:0007517 | Muscle organ development | 0.000222 | 0.007316 | 46 |
| GO:0001837 | Epithelial to mesenchymal transition | 0.0002727 | 0.008796 | 47 |
| GO:0030509 | BMP signaling pathway | 0.0003009 | 0.009503 | 48 |
| GO:0007005 | Mitochondrion organization | 0.0003096 | 0.009577 | 49 |
| GO:0007601 | Visual perception | 0.0003263 | 0.009809 | 50 |
| GO:0055093 | Response to hyperoxia | 0.00033 | 0.009809 | 51 |
| GO:0001569 | Patterning of blood vessels | 0.0003939 | 0.010748 | 52 |
| GO:0034641 | Cellular nitrogen compound metabolic process | 0.0004086 | 0.010748 | 53 |
| GO:0034375 | High-density lipoprotein particle remodeling | 0.0004173 | 0.010748 | 54 |
| GO:0007596 | Blood coagulation | 0.0004177 | 0.010748 | 55 |
| GO:0003209 | Cardiac atrium morphogenesis | 0.0004325 | 0.010748 | 56 |
| GO:0003256 | Regulation of transcription from RNA polymerase II promoter involved in myocardial precursor cell differentiation | 0.0004325 | 0.010748 | 57 |
| GO:0003273 | Cell migration involved in endocardial cushion formation | 0.0004325 | 0.010748 | 58 |
| GO:0035793 | Positive regulation of metanephric mesenchymal cell migration by platelet-derived growth factor receptor-beta signaling pathway | 0.0004325 | 0.010748 | 59 |
| GO:0072144 | Glomerular mesangial cell development | 0.0004325 | 0.010748 | 60 |
| GO:0086001 | Regulation of cardiac muscle cell action potential | 0.0004325 | 0.010748 | 61 |
| GO:0030512 | Negative regulation of transforming growth factor beta receptor signaling pathway | 0.0004426 | 0.010821 | 62 |
| GO:0007584 | Response to nutrient | 0.000549 | 0.013211 | 63 |
| GO:0032981 | Mitochondrial respiratory chain complex I assembly | 0.000634 | 0.014786 | 64 |
| GO:0086091 | Regulation of heart rate by cardiac conduction | 0.000634 | 0.014786 | 65 |
| GO:0007165 | Signal transduction | 0.0007557 | 0.017308 | 66 |
| GO:0043691 | Reverse cholesterol transport | 0.0007649 | 0.017308 | 67 |
| GO:0046777 | Protein autophosphorylation | 0.000813 | 0.017987 | 68 |
| GO:0048661 | Positive regulation of smooth muscle cell proliferation | 0.0008207 | 0.017987 | 69 |
| GO:0003344 | Pericardium morphogenesis | 0.0008601 | 0.017987 | 70 |
| GO:0043615 | Astrocyte cell migration | 0.0008601 | 0.017987 | 71 |
| GO:0060732 | Positive regulation of inositol phosphate biosynthetic process | 0.0008601 | 0.017987 | 72 |
| GO:0006184 | GTP catabolic process | 0.0008661 | 0.017987 | 73 |
| GO:0010508 | Positive regulation of autophagy | 0.0009121 | 0.018438 | 74 |
| GO:0035924 | Cellular response to vascular endothelial growth factor stimulus | 0.0009121 | 0.018438 | 75 |
| GO:0034329 | Cell junction assembly | 0.0009281 | 0.018513 | 76 |
| GO:0000122 | Negative regulation of transcription from RNA polymerase II promoter | 0.0009844 | 0.019382 | 77 |
| GO:0008015 | Blood circulation | 0.0010782 | 0.020956 | 78 |
| GO:0016477 | Cell migration | 0.00114 | 0.021708 | 79 |
| GO:0006915 | Apoptotic process | 0.0011455 | 0.021708 | 80 |
| GO:0071456 | Cellular response to hypoxia | 0.0011788 | 0.022062 | 81 |
| GO:0007220 | Notch receptor processing | 0.0012583 | 0.022983 | 82 |
| GO:0060326 | Cell chemotaxis | 0.0012583 | 0.022983 | 83 |
| GO:0014068 | Positive regulation of phosphatidylinositol 3-kinase cascade | 0.0012788 | 0.02308 | 84 |
| GO:0002576 | Platelet degranulation | 0.0013216 | 0.023571 | 85 |
| GO:0008283 | Cell proliferation | 0.0013726 | 0.024196 | 86 |
| GO:0006457 | Protein folding | 0.0014041 | 0.024279 | 87 |
| GO:0060666 | Dichotomous subdivision of terminal units involved in salivary gland branching | 0.0014254 | 0.024279 | 88 |
| GO:0086064 | Cell communication by electrical coupling involved in cardiac conduction | 0.0014254 | 0.024279 | 89 |
| GO:0060412 | Ventricular septum morphogenesis | 0.0014588 | 0.024572 | 90 |
| GO:0002027 | Regulation of heart rate | 0.0016785 | 0.027659 | 91 |
| GO:0010718 | Positive regulation of epithelial to mesenchymal transition | 0.0016785 | 0.027659 | 92 |
| GO:0008284 | Positive regulation of cell proliferation | 0.0018004 | 0.029348 | 93 |
| GO:0006955 | Immune response | 0.0019114 | 0.030827 | 94 |
| GO:0007044 | Cell-substrate junction assembly | 0.002126 | 0.032555 | 95 |
| GO:0032964 | Collagen biosynthetic process | 0.002126 | 0.032555 | 96 |
| GO:0046826 | Negative regulation of protein export from nucleus | 0.002126 | 0.032555 | 97 |
| GO:0051271 | Negative regulation of cellular component movement | 0.002126 | 0.032555 | 98 |
| GO:0086002 | Regulation of cardiac muscle cell action potential involved in contraction | 0.002126 | 0.032555 | 99 |
| GO:0045893 | Positive regulation of transcription, DNA-dependent | 0.002335 | 0.035399 | 100 |
| GO:0000278 | Mitotic cell cycle | 0.0024578 | 0.03656 | 101 |
| GO:0001937 | Negative regulation of endothelial cell proliferation | 0.0024598 | 0.03656 | 102 |
| GO:0018108 | Peptidyl-tyrosine phosphorylation | 0.0025167 | 0.036686 | 103 |
| GO:0097193 | Intrinsic apoptotic signaling pathway | 0.0025167 | 0.036686 | 104 |
| GO:0001649 | Osteoblast differentiation | 0.0026923 | 0.038871 | 105 |
| GO:0061418 | Regulation of transcription from RNA polymerase II promoter in response to hypoxia | 0.0027632 | 0.039357 | 106 |
| GO:0071260 | Cellular response to mechanical stimulus | 0.0028762 | 0.039357 | 107 |
| GO:0007507 | Heart development | 0.0029595 | 0.039357 | 108 |
| GO:0003208 | Cardiac ventricle morphogenesis | 0.0029596 | 0.039357 | 109 |
| GO:0006108 | Malate metabolic process | 0.0029596 | 0.039357 | 110 |
| GO:0009404 | Toxin metabolic process | 0.0029596 | 0.039357 | 111 |
| GO:0019800 | Peptide cross-linking via chondroitin 4-sulfate glycosaminoglycan | 0.0029596 | 0.039357 | 112 |
| GO:0061298 | Retina vasculature development in camera-type eye | 0.0029596 | 0.039357 | 113 |
| GO:0090051 | Negative regulation of cell migration involved in sprouting angiogenesis | 0.0029596 | 0.039357 | 114 |
| GO:0042594 | Response to starvation | 0.0030891 | 0.0407 | 115 |
| GO:0016568 | Chromatin modification | 0.0031143 | 0.0407 | 116 |
| GO:0043065 | Positive regulation of apoptotic process | 0.0031884 | 0.041312 | 117 |
| GO:0007528 | Neuromuscular junction development | 0.003438 | 0.043799 | 118 |
| GO:0072593 | Reactive oxygen species metabolic process | 0.003438 | 0.043799 | 119 |
| GO:0032355 | Response to estradiol stimulus | 0.0036998 | 0.045409 | 120 |
| GO:0030168 | Platelet activation | 0.0038859 | 0.045409 | 121 |
| GO:0006554 | Lysine catabolic process | 0.0039239 | 0.045409 | 122 |
| GO:0016264 | Gap junction assembly | 0.0039239 | 0.045409 | 123 |
| GO:0035313 | Wound healing, spreading of epidermal cells | 0.0039239 | 0.045409 | 124 |
| GO:0051895 | Negative regulation of focal adhesion assembly | 0.0039239 | 0.045409 | 125 |
| GO:0061314 | Notch signaling involved in heart development | 0.0039239 | 0.045409 | 126 |
| GO:0071372 | Cellular response to follicle-stimulating hormone stimulus | 0.0039239 | 0.045409 | 127 |
| GO:0086069 | Bundle of His cell to Purkinje myocyte communication | 0.0039239 | 0.045409 | 128 |
| GO:0090280 | Positive regulation of calcium ion import | 0.0039239 | 0.045409 | 129 |
| GO:2000573 | Positive regulation of DNA biosynthetic process | 0.0039239 | 0.045409 | 130 |
| GO:2000810 | Regulation of tight junction assembly | 0.0039239 | 0.045409 | 131 |
| GO:0015031 | Protein transport | 0.0040073 | 0.046023 | 132 |
| GO:0055114 | Oxidation-reduction process | 0.0040461 | 0.04612 | 133 |
| GO:0001701 | In utero embryonic development | 0.0041054 | 0.046446 | 134 |
| GO:0030513 | Positive regulation of BMP signaling pathway | 0.0042075 | 0.046901 | 135 |
| GO:0070301 | Cellular response to hydrogen peroxide | 0.0042075 | 0.046901 | 136 |
| GO:0007010 | Cytoskeleton organization | 0.0043977 | 0.048506 | 137 |
| GO:0007169 | Transmembrane receptor protein tyrosine kinase signaling pathway | 0.0044155 | 0.048506 | 138 |
