# Supplementary material for: Microarray expression profile of mRNAs and long noncoding RNAs and the potential role of PFK-1 in infantile hemangioma
Source: Cell Div. 2021 Jan 11;16:1. doi: 10.1186/s13008-020-00069-y (PMC7802351; doi:10.1186/s13008-020-00069-y)
Supplement: Supplementary file 6 — Additional file 6: Table S6. KEGG pathway analysis of differentially expressed mRNAs. [file 13008_2020_69_MOESM6_ESM.docx]

**Table S6.** KEGG pathway analysis of differentially expressed mRNAs.

| **Pathway ID** | **Pathway Name** | **P-value** | **FDR** | **Rank** |
| --- | --- | --- | --- | --- |
| 4510 | Focal adhesion | 4.61E-14 | 8.39E-12 | 1 |
| 1100 | Metabolic pathways | 7.93E-09 | 7.22E-07 | 2 |
| 4151 | PI3K-Akt signaling pathway | 1.01E-07 | 6.13E-06 | 3 |
| 4810 | Regulation of actin cytoskeleton | 4.16E-07 | 1.89E-05 | 4 |
| 5012 | Parkinson's disease | 5.31E-07 | 1.93E-05 | 5 |
| 5412 | Arrhythmogenic right ventricular cardiomyopathy (ARVC) | 3.96E-06 | 0.00012 | 6 |
| 280 | Valine, leucine and isoleucine degradation | 1.68E-05 | 0.000436 | 7 |
| 4670 | Leukocyte transendothelial migration | 2.01E-05 | 0.000439 | 8 |
| 4512 | ECM-receptor interaction | 2.17E-05 | 0.000439 | 9 |
| 4270 | Vascular smooth muscle contraction | 3.41E-05 | 0.000621 | 10 |
| 4974 | Protein digestion and absorption | 0.000101 | 0.001677 | 11 |
| 5010 | Alzheimer's disease | 0.000159 | 0.002258 | 12 |
| 5203 | Viral carcinogenesis | 0.000161 | 0.002258 | 13 |
| 5134 | Legionellosis | 0.000215 | 0.002783 | 14 |
| 5166 | HTLV-I infection | 0.000229 | 0.002783 | 15 |
| 5200 | Pathways in cancer | 0.000264 | 0.003001 | 16 |
| 190 | Oxidative phosphorylation | 0.000307 | 0.003189 | 17 |
| 5205 | Proteoglycans in cancer | 0.000319 | 0.003189 | 18 |
| 5034 | Alcoholism | 0.000339 | 0.003189 | 19 |
| 5016 | Huntington's disease | 0.000379 | 0.003189 | 20 |
| 4120 | Ubiquitin mediated proteolysis | 0.000386 | 0.003189 | 21 |
| 5322 | Systemic lupus erythematosus | 0.000386 | 0.003189 | 22 |
| 5031 | Amphetamine addiction | 0.000674 | 0.005332 | 23 |
| 5146 | Amoebiasis | 0.000714 | 0.005416 | 24 |
| 4520 | Adherens junction | 0.000819 | 0.005745 | 25 |
| 620 | Pyruvate metabolism | 0.000821 | 0.005745 | 26 |
| 71 | Fatty acid degradation | 0.001078 | 0.007008 | 27 |
| 2010 | ABC transporters | 0.001078 | 0.007008 | 28 |
| 4330 | Notch signaling pathway | 0.001505 | 0.009443 | 29 |
| 5410 | Hypertrophic cardiomyopathy (HCM) | 0.001646 | 0.009984 | 30 |
| 5222 | Small cell lung cancer | 0.001735 | 0.010188 | 31 |
| 4360 | Axon guidance | 0.001886 | 0.010617 | 32 |
| 5132 | Salmonella infection | 0.001925 | 0.010617 | 33 |
| 4530 | Tight junction | 0.00212 | 0.011349 | 34 |
| 5414 | Dilated cardiomyopathy | 0.002238 | 0.011639 | 35 |
| 4144 | Endocytosis | 0.003886 | 0.019645 | 36 |
| 20 | Citrate cycle (TCA cycle) | 0.004207 | 0.020696 | 37 |
| 4060 | Cytokine-cytokine receptor interaction | 0.004432 | 0.021226 | 38 |
| 5211 | Renal cell carcinoma | 0.004942 | 0.023064 | 39 |
| 640 | Propanoate metabolism | 0.005076 | 0.023095 | 40 |
| 4115 | p53 signaling pathway | 0.005511 | 0.024463 | 41 |
| 4720 | Long-term potentiation | 0.006445 | 0.027929 | 42 |
| 5416 | Viral myocarditis | 0.007482 | 0.031667 | 43 |
| 260 | Glycine, serine and threonine metabolism | 0.007706 | 0.031874 | 44 |
| 4260 | Cardiac muscle contraction | 0.008626 | 0.034888 | 45 |
| 5202 | Transcriptional misregulation in cancer | 0.009271 | 0.036682 | 46 |
| 4020 | Calcium signaling pathway | 0.010038 | 0.038872 | 47 |
| 4146 | Peroxisome | 0.010328 | 0.039158 | 48 |
| 5168 | Herpes simplex infection | 0.011419 | 0.042413 | 49 |
| 4640 | Hematopoietic cell lineage | 0.01382 | 0.050304 | 50 |
| 4010 | MAPK signaling pathway | 0.014545 | 0.051904 | 51 |
| 3015 | mRNA surveillance pathway | 0.016127 | 0.056446 | 52 |
| 310 | Lysine degradation | 0.01695 | 0.058205 | 53 |
| 5323 | Rheumatoid arthritis | 0.01737 | 0.058545 | 54 |
| 4145 | Phagosome | 0.023389 | 0.077395 | 55 |
| 500 | Starch and sucrose metabolism | 0.024414 | 0.079344 | 56 |
| 4114 | Oocyte meiosis | 0.031427 | 0.100345 | 57 |
| 4320 | Dorso-ventral axis formation | 0.035354 | 0.109059 | 58 |
| 4977 | Vitamin digestion and absorption | 0.035354 | 0.109059 | 59 |
| 5214 | Glioma | 0.036379 | 0.11035 | 60 |
| 5152 | Tuberculosis | 0.042372 | 0.126422 | 61 |
| 3320 | PPAR signaling pathway | 0.045867 | 0.132505 | 62 |
| 5218 | Melanoma | 0.045867 | 0.132505 | 63 |
| 760 | Nicotinate and nicotinamide metabolism | 0.047352 | 0.134658 | 64 |
