# Supplementary figures and images for: Microarray expression profile of mRNAs and long noncoding RNAs and the potential role of PFK-1 in infantile hemangioma
Source: Cell Div. 2021 Jan 11;16:1. doi: 10.1186/s13008-020-00069-y (PMC7802351; doi:10.1186/s13008-020-00069-y)

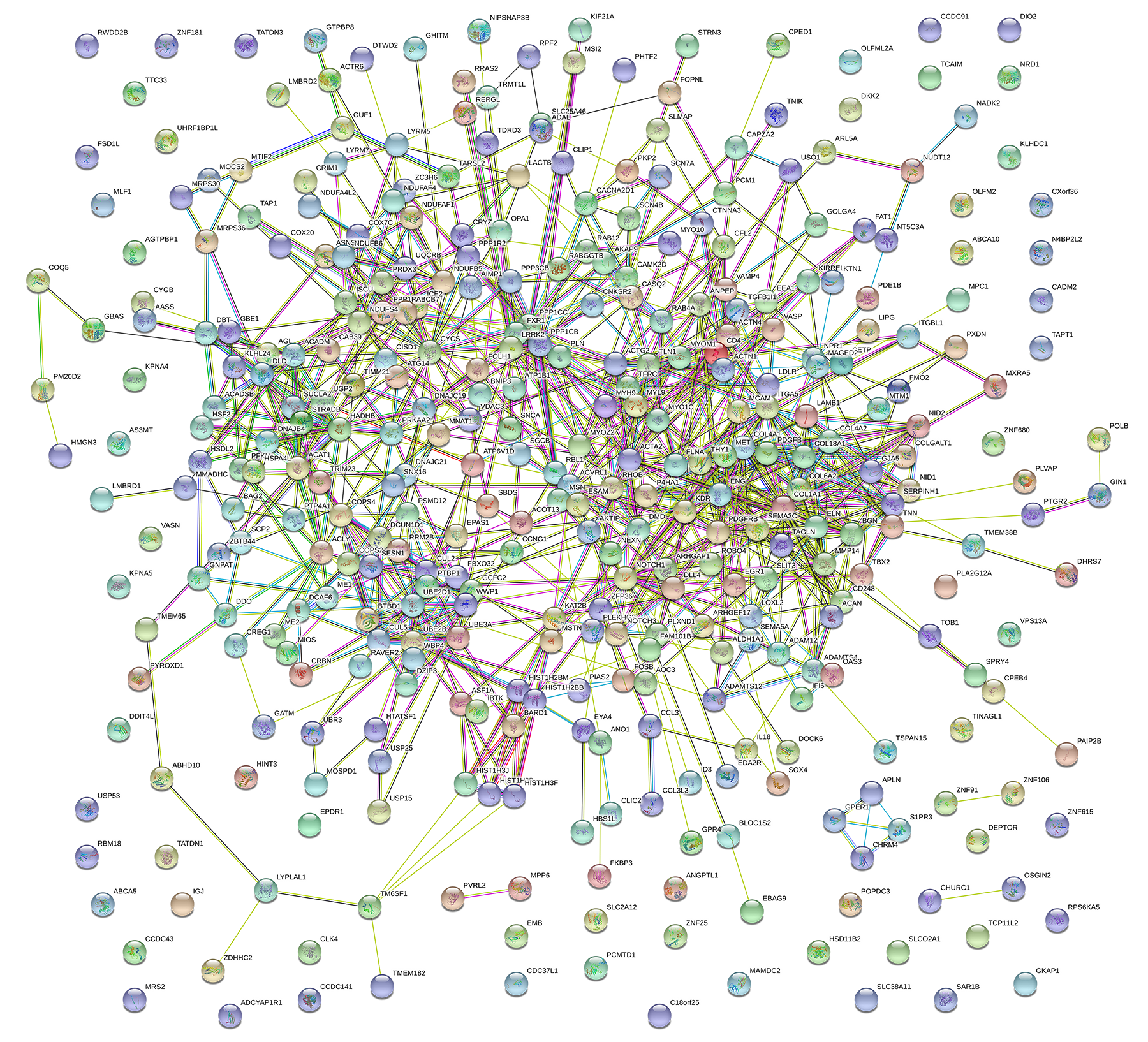

Supplement: Supplementary file 7 — Additional file 7: Figure S1. Protein-protein interaction network analysis by STRING. [file 13008_2020_69_MOESM7_ESM.tif]
